# Estimation of Ultrasonic Velocity, Density, Internal Pressure, and Thermophysical Parameters of Ionic Liquid Mixtures: Application of Flory’s Statistical Theory

**DOI:** 10.1021/acsomega.4c00520

**Published:** 2024-04-22

**Authors:** Archana Sirohi, Arun Upmanyu, Pankaj Kumar, Monika Dhiman, Kailash Chandra Juglan, Devinder Pal Singh, Kuldeep K. Saxena, Alok Bhadauria, Md Irfanul Haque Siddiqui

**Affiliations:** † Chitkara University Institute of Engineering and Technology, Chitkara University, Punjab 140401, India; ‡ Department of Physics, Lovely Faculty of Technology and Sciences, Lovely Professional University, Punjab 144001, India; § Acoustics Research Center, Mississauga L5A 1Y7, Ontario, Canada; ∥ Division of Research and Development, Lovely Professional University, Phagwara, Punjab 144001, India; ⊥ Department of Mechanical and Industrial Engineering, Manipal Institute of Technology Bengaluru, Manipal Academy of Higher Education, Manipal 576104, Karnataka, India; # Mechanical Engineering Department, College of Engineering, King Saud University, Riyadh 11451, Saudi Arabia

## Abstract

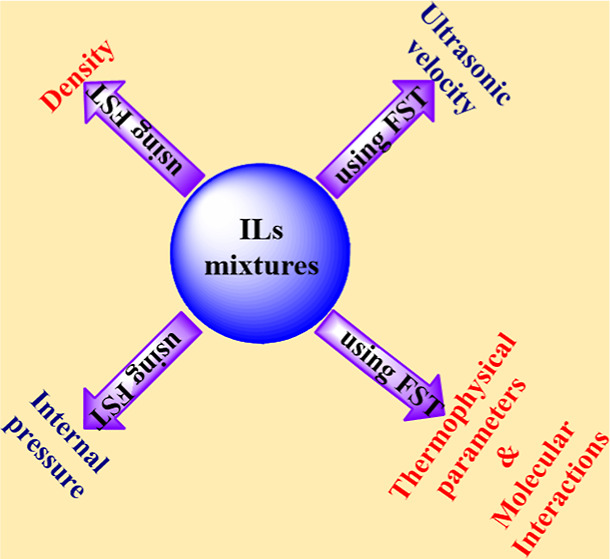

Flory’s statistical theory (FST) has been employed to estimate the ultrasonic velocity, density, internal pressure, and several important thermophysical parameters such as the energy of vaporization, the heat of vaporization, cohesive energy density, polarity index, and solubility for eight binary mixtures of ionic liquids and water within the temperature range of 288.15 to 308.15 K. The ionic liquids chosen for this investigation are [BMim][dca], [BMim][TfO], [BMpy][TfO], [BMpyr][dca], [BMpyr][TfO], [EEPy][ESO_4_], [HMim][dca], and [MPy][MSO_4_]. The predicted values of ultrasonic velocity and density show good agreement with the data reported in the literature. It endorses the applicability of FST to these binary mixtures. A comparative analysis of the internal pressure values (*P*_i_) determined by using FST and the standard thermodynamic approach is also presented. The results obtained for *P*_i_ using both approaches show good agreement. Besides, for the mixtures under study, the correlation between ultrasonic velocity, density, and surface tension has also been examined. The variation of thermophysical parameters with concentration and temperature changes has been utilized to explore the nature and strength of the solute–solvent interactions prevalent in these mixtures. It is pointed out that A–A-type interactions dominate over A-B-type interactions in water-rich regions of the mixtures.

## 1 Introduction

Ionic liquids (ILs) have received the attention of many researchers and industrialists due to their exceptional properties, such as low vapor pressure, a wide range of viscosity, adjustable miscibility, and good thermal conductivity.^1,2^ Another promising feature of ILs is their tunable physical and chemical properties. ILs are often termed designer solvents, which makes them extremely useful for special applications in industry. Ultrasonic velocity is one of the prime properties of ILs. It is used in the formulation of equations of state and to derive many thermophysical properties. Recently, some researchers^3,4^ calculated heat capacity, isothermal and adiabatic compressibility, molecular radius, and apparent isentropic compressibility for ILs and their mixtures using the speed of sound in conjunction with other thermophysical parameters. It is a matter of fact that for the industrial design processes of ILs, it is customary to determine their density and refractive index. Nowadays, density calculation is being used to solve the material or energy balance equations of chemical processes in industry.^5^

The study of molecular interactions is important in almost all fields of the physical and chemical sciences. These interactions provide valuable information about the molecular packing, orientation, and conformation of the molecules. Many researchers^6−9^ have investigated the molecular interactions in ILs and some binary liquid mixtures. Fumino et al.^10^ investigated the hydrogen bonding, Coulomb interactions, and dispersion forces in some ILs. Dhumal et al.^11^ reported the molecular interactions of a Cu-based metal–organic framework with a confined imidazolium-based IL using experimental and computational techniques. Recently, Wei et al.^12^ reported changes in molecular interactions in ILs with charged SiO_2_ surfaces. It is reported^13−15^ that the computational and experimental techniques are complementary for determining the structure, design, and thermophysical properties of liquids and their mixtures. Some researchers^16−18^ employed density functional theory (DFT), molecular dynamics (MD), COSMO-R, and Flory’s statistical theory (FST) to estimate these properties. Though all these theoretical formulations are found suitable to compute the thermophysical properties, FST is a valuable and powerful tool as a result of the limited input parameters and ease of calculations using a simple analytic expression. FST is a good candidate in the theoretical framework of industrial design to predict the thermodynamic properties.^19^ Several researchers^20−22^ successfully employed FST to predict the ultrasonic velocity and density with reasonable accuracy for pure liquids. Pandey et al.^23^ modified FST for ternary liquid mixtures to discuss their thermodynamic behavior. Oswal et al.^24^ tested the validity of the FST, ERAS, and Rao theories for the alkyl amines. Gepert et al.^25^ have compared FST and the Prigogine–Flory–Patterson (PFP) model for binary mixtures of hydrocarbons. Recently, Shrivastava et al.^26^ estimated various thermodynamic parameters using FST for pure ILs at elevated pressure. However, despite the high demand for ILs and their mixtures in various fields, their physicochemical properties have not yet been systematically studied, particularly for the mixtures of ILs with molecular liquids.^27^ Thus, there is a need to compute thermophysical properties like the speed of sound, density, adiabatic compressibility, thermal expansion, internal pressure, etc., for these liquids. Recently, binary mixtures of ILs and water have gained a lot of impetus.^28−30^ In their review article, Isosaari et al.,^31^ have summarized the use of ILs for wastewater treatment. These findings motivated us to conduct in-depth investigations of the physical properties of water-based IL mixtures.

In this investigation, FST is employed to estimate ultrasonic velocity, density, and internal pressure for eight binary mixtures of ILs and water at various temperatures. The obtained results are compared to the literature values, and a reasonable agreement is found between them. The correlation of density, ultrasonic velocity, and surface tension was also investigated. Several researchers have reported the importance of such correlations.^8,32^ Besides, some important thermophysical parameters were determined to understand the molecular interactions prevalent in these binary mixtures. The concentration and temperature dependence of solubility parameters and several other thermophysical parameters were also been investigated. The data required for the present study has been taken from the literature.^33^

## 2 Theoretical Formulation

FST formulations have been discussed at length by many workers.^34−37^ They have successfully employed it to compute the density (*d*), ultrasonic velocity (*U*), surface tension, and molar volume for organic liquids, polymers, and ILs. We extended the same formulation to IL mixtures. Herein, only those relations are reported that have been directly utilized in the present study.

Using a reduced equation of state,^38^ reduced volume (*Ṽ*), reduced temperature (*T̃*), characteristic pressure (P*), characteristic temperature (T*), and characteristic volume (V*) for pure ILs can be deduced as per the equations given below. Herein, the characteristic volume (V*) and characteristic temperature (T*) are the molar volume and temperature at the zero pressure limit (*P* = 0)^39^
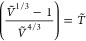
1where  and  are the reduced volume and reduced temperature, respectively.

The reduced volume *Ṽ* in terms of the coefficient of thermal expansion (α) can be computed using the relation given below:
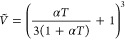
2where α can be calculated in terms of ultrasonic velocity (*U*) and density (*d*) at various temperatures and concentrations using a well-known relation available in the literature:
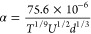
3P* can be evaluated from the knowledge of α and *K*_T_:
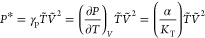
4where γ_P_ is the thermal pressure coefficient at the zero pressure limit and *K*_T_ is the isothermal compressibility. *K*_T_ was determined using a formula taken from the literature.^40,41^ The above relations have been employed to compute P*, V*, and T* for mixture components. Thereafter, these parameters were used to determine the segment fractions (ψ), site fraction (θ), and interaction parameter (χ_12_) for the binary mixtures. The segment fractions are calculated as follows:
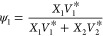
5and
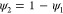
6where ψ_1_ and ψ_2_ are the segment fractions, *V*_i_* (*i* = 1, 2) is the characteristic volume, and *X*_*i*_ (*i* = 1, 2) is the mole fraction of solute (water) and solvent (ILs), respectively. The site fraction is given by
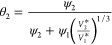
7The interaction parameter is calculated as
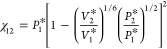
8The segment fractions (ψ_1_ and ψ_2_), site fraction (θ_2_), and interaction parameter (χ_12_) are used to determine the values of characteristic pressure (P*) and characteristic temperature (T*) of mixtures based on the following relations:
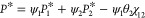
9
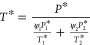
10

### 2.1 Surface Tension for the Binary Ionic Liquid’s Mixture and Flory Theory

Patterson and Rastogi^42^ derived the characteristic surface tension (σ*) and reduced surface tension [σ(*Ṽ*)] for pure liquids based upon the principle of corresponding states. They further used these parameters to calculate the surface tension; σ = σ* × σ(*Ṽ*). Some researchers^43^ extended the same formulation to compute surface tension for IL. In this study, we extended the same approach to a binary mixture of ILs and water.

The characteristic surface tension (σ*) for binary mixtures can be calculated as
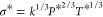
11where P* and T* are calculated using eqs 9 and 10 and *k* is the Boltzmann constant.

The reduced surface tension [σ(*Ṽ*)] is deduced as a function of the reduced volume (*Ṽ*_m_) for binary mixtures as
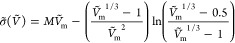
12where *M* is the fractional change in the neighborhood cell count in the surface phase. Its value generally exists between 0.26 and 0.29. The surface tension of an IL mixture is computed using the following equation:
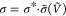
13

### 2.2 Estimation of Ultrasonic Velocity and Their Correlation with Density and Surface Tension

Ultrasonic velocity plays a vital role in chemical ultrasonics. Simply, in conjunction with density, it can provide valuable information about liquid systems. In the present study, the density (*d*) and a diagnostic parameter, the surface tension (σ), are utilized to calculate the ultrasonic velocity of these systems using the standard correlation available in the literature:^44,45^Auerbach relation:
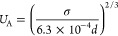
14Alternberg relation:
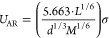
15Singh–Pandey–Sanguri relation:
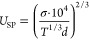
16modified Auerbach relation:
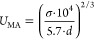
17

### 2.3 FST and Estimation of Density

The ideal reduced volume *Ṽ*^0^ of the binary mixture can be obtained as
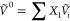
18*Ṽ*^0^ is further used to calculate the ideal reduced temperature, *T̃*^0^:
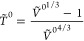
19The excess reduced volume is given by
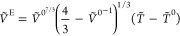
20The reduced volume is then obtained as
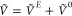
21Finally, the molar volume (*V*_m_) in terms of the characteristic volume of the mixture (V*) and the reduced volume (calculated using eq 21) can be obtained as
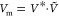
22V* can be calculated using the additive properties of the mixture:
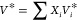
23The density of the mixture (*d*) is then given by
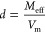
24where *M*_eff_ is the effective mass of the mixture and *V*_m_ is calculated using eq 22.

### 2.4 Estimation of Internal Pressure and FST

Internal pressure is a very useful thermophysical parameter, and its importance in the field of thermodynamics was first highlighted by Hildebrand and later by various workers.^46−50^ Due to the complex procedure of its experimental measurements, many empirical/semiempirical relations have been proposed to compute the internal pressure based on ultrasonic velocity, molecular radius, and thermal expansion at different temperatures for pure as well as mixtures. The thermodynamic method is the most prominent approach to computing the internal pressure (*P*_i_), which is rewritten below in its usual form:
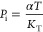
25where α and *K*_T_ are the thermal expansivity and isothermal compressibility of the given mixtures, which can be deduced using the following standard relations:^51^
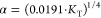
26
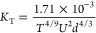
27

Pandey and co-workers computed the internal pressure (*P*_FST_) for liquid mixtures at 303.15 K using FST. Later, the same approach was opted by many workers to compute the internal pressure for different liquid mixtures. The mathematical formulation to compute the *P*_FST_ is reproduced below:
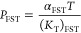
28

In the above relation, α_FST_ and (*K*_T_)_FST_ are the thermal expansivity and isothermal compressibility calculated using Flory’s parameters as
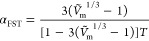
29
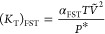
30In the above relations, *Ṽ*_m_ and P* are the reduced volume and characteristic pressure of mixtures; their values have been computed using eqs 2 and 9. Herein, we have also employed eq 28 for the first time to compute the *P*_*i*_ values for the binary mixtures of water and ILs. The obtained results are then compared with the *P*_*i*_ values obtained by using the thermodynamic method.

### 2.5 Application of FST to Compute the Thermophysical Parameters of IL Mixtures

In this work, FST has been employed for the first time to compute some important thermodynamic parameters,^32^ viz., energy of vaporization (Δ*E*_V_), heat of vaporization (Δ*H*), cohesive energy density (ced), solubility parameter (δ), and polarity index (*n*) for binary mixtures of water and ILs, using the following relations:
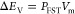
31
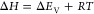
32

The cohesive energy density basically depends upon Δ*E*_V_, *n*, and the molar volume (*V*_m_) of the mixture. The general form of the relation is given below:
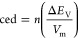
33where here the polarity index is *n* = 1. The solubility parameter is given by
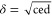
34Hildebrand originally developed the solubility parameter relations for organic liquids. Here, we are investigating its application to binary mixtures of water and ILs.

## 3 Results and Discussion

### 3.1 Estimation of Ultrasonic Velocity Using FST

In the present study, ultrasonic velocity (*U*) is computed for eight binary mixtures of water and ILs using four empirical relations, viz., Auerbach relation (*U*_A_), Alternberg relation (*U*_AR_), Singh–Pandey–Sanguri relation (*U*_SR_), and modified Auerbach relation (*U*_MR_) (eqs 14–17). The following ILs were used in the present investigation.

I. 1-Butyl-3-methylimidazolium dicyanamide [BMim][dca]
II. 1-Butyl-3-methylimidazolium trifluoromethanesulfonate [BMim][TfO]
III. 1-Butyl-3-methylpyridinium trifluoromethanesulfonate [BMpy][TfO]
IV. 1-Butyl-1-methylpyrrolidinium dicyanamide [BMpyr][dca]
V. 1-Butyl-1-methylpyrrolidinium trifluoromethanesulfonate [BMpyr][TfO]
VI. 1,2-Diethylpyridinium ethylsulfate [EEPy][ESO_4_]
VII. 1-Hexyl-3-methylimidazolium dicyanamide [HMim][dca]
VIII. 1-Methylpyridinium methylsulfate [MPy][MSO_4_]

**Figure 1:**
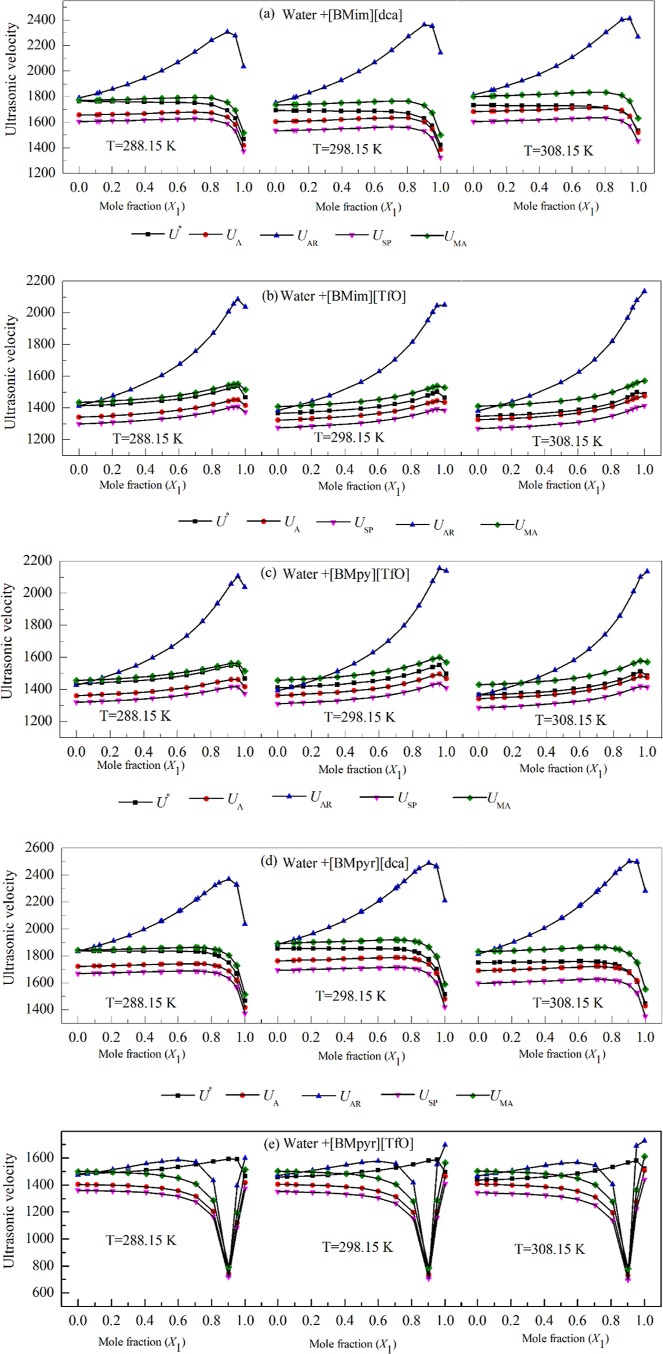

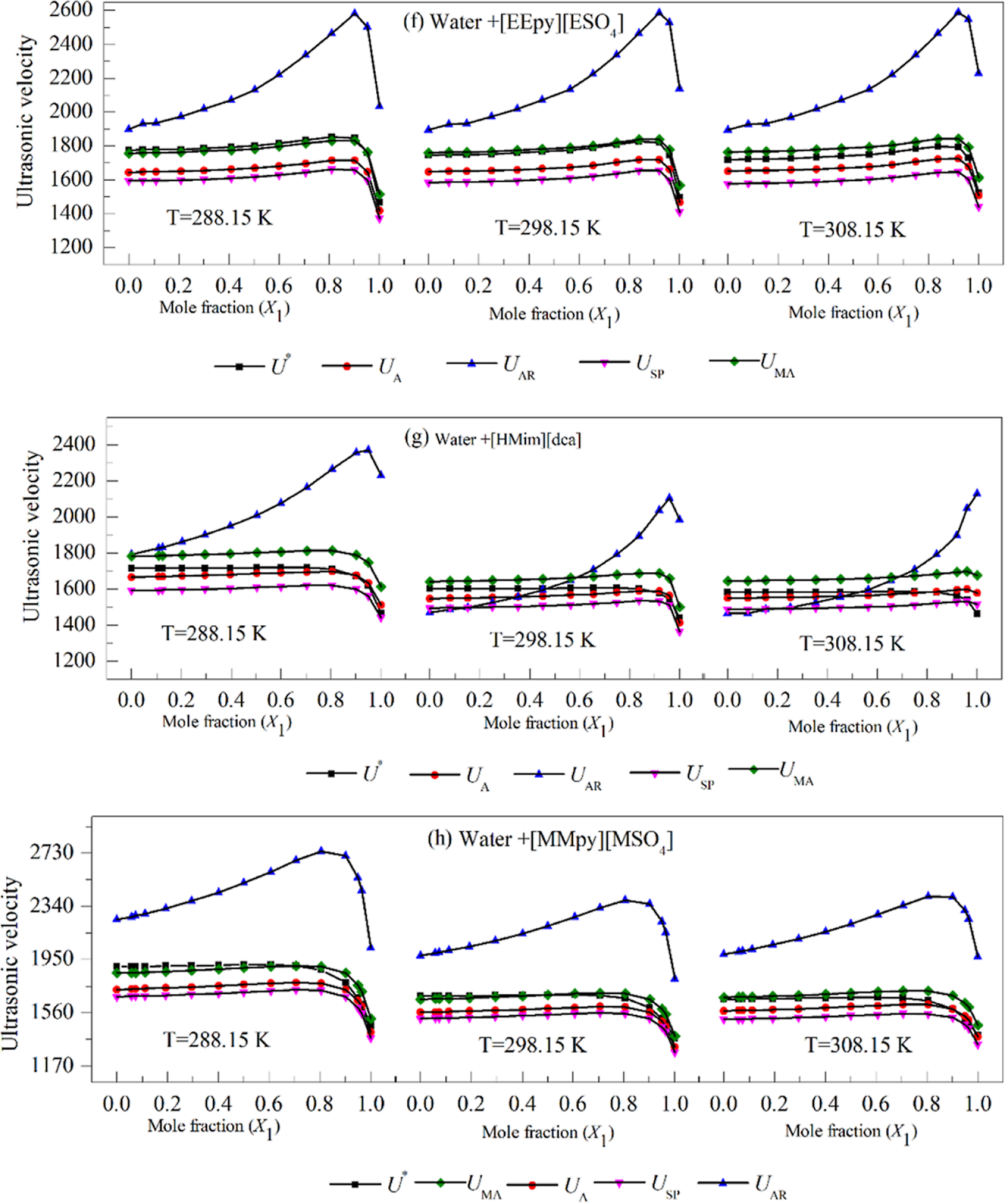
Comparison of ultrasonic velocity estimated using the Auerbach relation (*U*_A_), Alternberg relation (*U*_AR_), Singh–Pandey–Sanguri relation (*U*_SP_), and modified Auerbach relation (*U*_MA_) along with the literature values (U*) for binary mixtures. Solid line is a guide for eye.

Theoretically obtained values of the ultrasonic velocity are reported in Table S1 (Supporting Information) as well as plotted against the mole fraction (*X*_1_) of water at different temperatures and depicted in Figure 1a–h. The close look of Figure 1a indicates that for water + [BMim][dca] binary mixture, the *U* values obtained using Auerbach relation (*U*_A_), Singh–Pandey–Sanguri relation (*U*_SP_), and modified Auerbach relation (*U*_MA_) are in reasonable agreement with ultrasonic velocity (U*) data reported in the literature.^33^ However, the Alternberg relation (*U*_AR_) reports a strong deviation with the increase in the mole fraction of water. This deviation is much more pronounced for the higher mole fractions of water. Figure 1b–h also indicates similar results, which confirm that except for *U*_AR_, the other three relations; *U*_A_, *U*_MA_, and *U*_SP_, can be employed to compute the ultrasonic velocity for water + [BMim][TfO], water + [BMpy][TfO], water + [BMpyr][dca], water + [EEpy][ESO_4_], water + [HMim][dca], and water + [MMpy][MSO_4_] mixtures. In the case of water+[BMpyr][TfO] [Figure 1e], the deviation of *U*_A_, *U*_MA_, and *U*_SP_ with respect to U* increased for *X*_1_ > 0.7. It is also noticed for the same system that the Alternberg relation (*U*_AR_) shows reasonable agreement until *X*_1_ = 0.7.

The reasonable agreement of *U*_A_, *U*_MA_, and *U*_SP_ with U* also affirms the *U*–*d*–*σ* correlation and the ability of FST to compute the ultrasonic velocity for the given water + IL mixtures. The higher deviation of *U*_AR_ values from the literature data is due to the noncompliance of the assumptions made for the derivation of this relation. The general agreement of *U*_A_, *U*_MA_, and *U*_SP_ with U* is further confirmed from the absolute percentage deviation (Table 1). It is pertinent to mention here that, however, the general agreement is reached between theoretical models (*U*_A_, *U*_MA_, and *U*_SP_) with U*, but the deviation is still toward the higher side. It is attributed to the fact that these relations were originally derived for organic mixtures having component size is almost similar, but in the present systems, the size and structure of ILs and water molecules are different, which leads to a higher deviation. It further suggests that these relations also need modification, considering the complex structure of IL to estimate the ultrasonic velocity for given mixtures.

**Table 1:** Absolute Percentage Deviation of Computed Ultrasonic Velocity from Experimental Values for Eight Ionic Liquid Mixtures

*T*/K	*U*_A_	*U*_AT_	*U*_SP_	*U*_MA_	*T*/K	*U*_A_	*U*_AT_	*U*_SP_	*U*_MA_
	Water + [BMim][dca]					Water + [BMpyr][TfO]			
288.15	4.63	17.83	7.59	1.95	288.15	5.47	14.57	8.4	1.05
298.15	3	19.86	6.72	3.7	298.15	5.47	14.57	8.4	1.05
308.15	1.45	21.87	5.83	5.46	308.15	2.14	18.36	6.58	4.61
	Water + [BMim][TfO]					Water + [EEpy][ESO4]			
288.15	4.95	17.43	7.9	1.6	288.15	2.14	18.36	6.58	4.61
298.15	3.31	19.48	7.02	3.36	298.15	7.09	20.2	9.97	1.19
308.15	1.65	21.48	6.11	5.13	308.15	3.79	24.32	8.16	2.85
	Water + [BMpy][TfO]					Water + [HMim][dca]			
288.15	5.13	15.93	8.07	1.42	288.15	1.45	21.87	5.83	5.46
298.15	3.31	16.33	7.02	3.36	298.15	2.55	14.85	5.95	4.55
308.15	1.65	18.02	6.11	5.14	308.15	1.5	15.43	5.09	6.28
	Water + [BMpyr][dca]					Water + [Mpy][MSO4]			
288.15	4.74	20.62	7.69	1.83	288.15	6.31	33.4	9.21	2.49
298.15	4.74	20.62	7.69	1.83	298.15	6.31	33.4	9.21	2.49
308.15	1.54	24.7	5.99	5.28	308.15	3.69	38.06	7.63	3.43

### 3.2 Estimation of Density Using FST

Density is an important thermodynamic parameter. It is presently used to characterize liquid mixtures in industry.^19,38,52^ It is also used in conjunction with ultrasonic velocity and temperature to compute important physical parameters, such as the coefficient of thermal expansion. In the present study, the density (*d*_FST_) of eight binary IL mixtures is computed using FST, and the obtained results are compared with the literature values (d*). Density data, computed by using FST, and their percentage deviation from the literature values at various concentrations and temperatures are reported in Table 2. A perusal of Table 2 reveals good agreement between the computed and literature values of density for all the systems under study. The mean percentage deviation (MPD) for each system at different temperatures is reported in Table 3. It is obvious from Table 3 that MPD is less than 1.5% for all the binary mixtures of ILs at all temperatures. It confirms the validity of FST to predict density in the given concentration and temperature range.

**Table 2:** Computed Density (*d*_*FST*_) and Literature (d*)^33^ Density along with Percentage Deviations for Eight Binary Mixtures of Water and Ionic Liquids at Different Temperatures^a^

*X*_1_	d*	*d*_FST_	% Dev	d*	*d*_FST_	% Dev	d*	*d*_FST_	% Dev
	*T* = 288.15 K			*T* = 298.15 K			*T* = 308.15 K		
Water + [BMim][dca]									
0	1066.58	1066.58	0.00	1060.17	1060.17	0.00	1053.83	1053.83	0.00
0.1088	1065.59	1067.14	0.15	1059.16	1060.71	0.15	1054.35	1052.79	0.15
0.1227	1065.44	1067.21	0.17	1059.01	1060.78	0.17	1054.42	1052.64	0.17
0.2017	1064.62	1067.68	0.29	1058.16	1061.23	0.29	1054.85	1051.77	0.29
0.2943	1063.46	1068.27	0.45	1056.98	1061.8	0.46	1055.39	1050.55	0.46
0.3952	1061.88	1068.97	0.67	1055.35	1062.45	0.67	1056.02	1048.88	0.68
0.503	1059.83	1069.7	0.93	1053.24	1063.14	0.94	1056.66	1046.7	0.95
0.5985	1057.37	1070.18	1.21	1050.71	1063.58	1.22	1057.06	1044.09	1.24
0.7032	1053.63	1070.06	1.56	1046.89	1063.42	1.58	1056.87	1040.15	1.61
0.805	1047.26	1067.76	1.96	1040.5	1061.18	1.99	1054.65	1033.69	2.03
0.9006	1035.92	1058.19	2.15	1029.6	1052	2.18	1045.77	1023.11	2.21
0.9503	1024.07	1043	1.85	1018.75	1037.62	1.85	1032.01	1013.06	1.87
1	999.1	999.1	0.00	997.04	997.04	0.00	994.02	994.02	0.00
Water + [BMim][TfO]									
0	1307.5	1307.5	0.00	1299.55	1299.55	0.00	1291.62	1291.62	0.00
0.1348	1305.34	1303.64	0.13	1297.37	1295.68	0.13	1289.44	1287.74	0.13
0.2028	1303.95	1301.3	0.20	1295.98	1293.3	0.21	1288.04	1285.33	0.21
0.2056	1303.89	1301.2	0.21	1295.92	1293.22	0.21	1287.97	1285.23	0.21
0.3083	1301.23	1296.7	0.35	1293.25	1288.66	0.36	1285.29	1280.65	0.36
0.4985	1285.26	1273.42	0.68	1277.25	1265.25	0.69	1269.28	1257.08	0.71
0.6062	1273.53	1258.59	0.93	1265.56	1250.44	0.95	1257.63	1242.22	0.97
0.7015	1249.11	1231.3	1.19	1241.35	1223.33	1.21	1233.61	1215.23	1.24
0.8064	1198.93	1180.1	1.45	1191.87	1172.98	1.47	1184.71	1165.56	1.51
0.8998	1166.61	1149.84	1.60	1160.13	1143.39	1.61	1153.47	1136.56	1.64
0.9309	1130.07	1116.26	1.46	1124.36	1110.62	1.46	1118.34	1104.51	1.49
0.9548	999.1	999.1	1.24	997.04	997.04	1.24	994.02	994.02	1.25
1	1285.26	1273.42	0.00	1277.25	1265.25	0.00	1269.28	1257.08	0.00
Water + [BMpy][TfO]									
0	1287.07	1287.07	0.00	1279.41	1279.41	0.00	1271.8	1271.8	0.00
0.0794	1286.06	1285.23	0.06	1278.62	1277.62	0.08	1271	1269.98	0.08
0.1517	1284.96	1283.28	0.13	1277.76	1275.63	0.17	1270.13	1267.96	0.17
0.2502	1283.1	1279.99	0.24	1276.28	1272.28	0.31	1268.64	1264.58	0.32
0.3515	1280.54	1275.76	0.38	1274.23	1268	0.49	1266.57	1260.24	0.50
0.4528	1276.96	1270.21	0.53	1271.33	1262.4	0.71	1263.65	1254.58	0.72
0.5628	1271.08	1261.9	0.73	1266.49	1254.01	1.00	1258.79	1246.08	1.02
0.6561	1263.08	1251.55	0.92	1259.79	1243.59	1.30	1252.08	1235.57	1.34
0.7494	1249.41	1235.48	1.13	1248.09	1227.52	1.68	1240.41	1219.46	1.72
0.8383	1224.23	1208.29	1.32	1225.8	1200.56	2.10	1218.28	1192.69	2.15
0.9201	1171.21	1155.76	1.34	1176.4	1149.07	2.38	1169.47	1142.04	2.40
0.9603	1114.93	1103.57	1.03	1120.78	1098.18	2.06	1114.78	1092.3	2.06
1	999.1	999.1	0.00	997.04	997.04	0.00	994.02	994.02	0.00
Water + [BMpyr][dca]									
0	1019.17	1019.17	0.00	1019.17	1019.17	0.00	1007.93	1007.93	0.00
0.096	1020.09	1018.92	0.11	1020.09	1018.92	0.11	1008.81	1007.63	0.12
0.1277	1020.42	1018.84	0.16	1020.42	1018.84	0.16	1009.13	1007.52	0.16
0.2116	1021.41	1018.6	0.28	1021.41	1018.6	0.28	1010.06	1007.23	0.28
0.3077	1022.73	1018.31	0.43	1022.73	1018.31	0.43	1011.31	1006.85	0.44
0.3942	1024.14	1018.08	0.60	1024.14	1018.08	0.60	1012.63	1006.5	0.61
0.4952	1026.08	1017.78	0.82	1026.08	1017.78	0.82	1014.45	1006.03	0.84
0.5023	1026.23	1017.76	0.83	1026.23	1017.76	0.83	1014.59	1005.99	0.86
0.6047	1028.62	1017.45	1.10	1028.62	1017.45	1.10	1016.84	1005.42	1.14
0.6137	1028.85	1017.41	1.12	1028.85	1017.41	1.12	1017.05	1005.35	1.16
0.7061	1031.35	1016.91	1.42	1031.35	1016.91	1.42	1019.41	1004.54	1.48
0.7177	1031.68	1016.81	1.46	1031.68	1016.81	1.46	1019.71	1004.4	1.52
0.7571	1032.76	1016.39	1.61	1032.76	1016.39	1.61	1020.75	1003.86	1.68
0.8208	1034.21	1015.22	1.87	1034.21	1015.22	1.87	1022.17	1002.57	1.95
0.8468	1034.51	1014.46	1.98	1034.51	1014.46	1.98	1022.5	1001.84	2.06
0.9034	1033.48	1011.96	2.13	1033.48	1011.96	2.13	1021.76	999.84	2.19
0.904	1033.44	1011.92	2.13	1033.44	1011.92	2.13	1021.73	999.81	2.19
0.9492	1027.84	1008.27	1.94	1027.84	1008.27	1.94	1017.1	997.62	1.95
0.9507	1027.5	1008.1	1.92	1027.5	1008.1	1.92	1016.81	997.53	1.93
0.9517	1027.26	1007.98	1.91	1027.26	1007.98	1.91	1016.62	997.47	1.92
1	999.1	999.1	0.00	999.1	999.1	0.00	994.02	994.02	0.00
Water + [BMpyr][TfO]									
0	1260.33	1260.33	0.00	1260.33	1260.33	0.00	1245.78	1245.78	0.00
0.0559	1259.79	1259.04	0.06	1259.79	1259.04	0.06	1245.23	1244.55	0.05
0.1084	1259.22	1257.8	0.11	1259.22	1257.8	0.11	1244.64	1243.3	0.11
0.2076	1257.91	1255.12	0.22	1257.91	1255.12	0.22	1243.3	1240.51	0.22
0.2997	1256.32	1252.05	0.34	1256.32	1252.05	0.34	1241.66	1237.3	0.35
0.408	1253.73	1247.41	0.51	1253.73	1247.41	0.51	1239.01	1232.47	0.53
0.5038	1250.37	1241.91	0.68	1250.37	1241.91	0.68	1235.59	1226.79	0.72
0.6003	1245.2	1234.21	0.89	1245.2	1234.21	0.89	1230.37	1218.89	0.94
0.7052	1235.58	1221.39	1.16	1235.58	1221.39	1.16	1220.74	1205.88	1.23
0.8095	1216.25	1198.77	1.46	1216.25	1198.77	1.46	1201.62	1183.37	1.54
0.9018	1174.84	1155.9	1.64	1174.84	1155.9	1.64	1161.16	1141.73	1.70
0.9533	1119.91	1104.38	1.41	1119.91	1104.38	1.41	1108.18	1092.63	1.42
1	999.1	999.1	0.00	999.1	999.1	0.00	994.02	994.02	0.00
Water + [EEpy][ESO_4_]									
0	1245.78	1245.78	0.00	1225.93	1225.93	0.00	1212.65	1212.65	0.00
0.0559	1245.23	1244.55	0.05	1225.38	1224.31	0.09	1212.07	1211.14	0.08
0.1084	1244.64	1243.3	0.11	1225.32	1224.06	0.10	1212.01	1210.87	0.09
0.2076	1243.3	1240.51	0.22	1224.62	1222.4	0.18	1211.26	1209.22	0.17
0.2997	1241.66	1237.3	0.35	1223.65	1219.81	0.31	1210.22	1206.65	0.30
0.408	1239.01	1232.47	0.53	1222.32	1216.82	0.45	1208.8	1203.43	0.45
0.5038	1235.59	1226.79	0.72	1220.31	1212.76	0.62	1206.68	1199.29	0.62
0.6003	1230.37	1218.89	0.94	1216.37	1205.78	0.88	1202.6	1192.05	0.89
0.7052	1220.74	1205.88	1.23	1209.02	1194.96	1.18	1195.12	1180.85	1.21
0.8095	1201.62	1183.37	1.54	1195.41	1177.39	1.53	1181.48	1163.05	1.58
0.9018	1161.16	1141.73	1.70	1161.14	1138.4	2.00	1147.67	1124.48	2.06
0.9533	1108.18	1092.63	1.42	1116.26	1092.23	2.20	1104.07	1080.72	2.16
1	994.02	994.02	0.00	999.1	999.1	0.00	994.02	994.02	0.00
Water + [HMim][dca]									
0	1053.83	1053.83	0.00	1028.56	1028.56	0.00	1022.4	1022.4	0.00
0.1088	1054.35	1052.79	0.15	1029.11	1028.25	0.08	1022.95	1022.07	0.09
0.1227	1054.42	1052.64	0.17	1029.43	1028.07	0.13	1023.26	1021.88	0.13
0.2017	1054.85	1051.77	0.29	1030.26	1027.61	0.26	1024.07	1021.38	0.26
0.2943	1055.39	1050.55	0.46	1031.25	1027.08	0.41	1025.04	1020.81	0.41
0.3952	1056.02	1048.88	0.68	1032.49	1026.41	0.59	1026.26	1020.08	0.61
0.503	1056.66	1046.7	0.95	1034.24	1025.46	0.86	1027.97	1019.04	0.88
0.5985	1057.06	1044.09	1.24	1036.25	1024.31	1.17	1029.93	1017.78	1.19
0.7032	1056.87	1040.15	1.61	1039	1022.4	1.62	1032.63	1015.71	1.67
0.805	1054.65	1033.69	2.03	1041.88	1019.6	2.19	1035.45	1012.81	2.24
0.9006	1045.77	1023.11	2.21	1043.09	1014.13	2.86	1036.72	1007.62	2.89
0.9503	1032.01	1013.06	1.87	1038.1	1009.12	2.87	1032.04	1003.34	2.86
1	994.02	994.02	0.00	997.04	997.04	0.00	994.02	994.02	0.00
Water + [Mpy][MSO_4_]									
0	1352.86	1352.86	0.00	1352.86	1352.86	0.00	1339.37	1339.37	0.00
0.0574	1351.6	1350.36	0.09	1351.6	1350.36	0.09	1338.1	1336.86	0.09
0.073	1351.23	1349.61	0.12	1351.23	1349.61	0.12	1337.74	1336.11	0.12
0.1111	1350.29	1347.74	0.19	1350.29	1347.74	0.19	1336.78	1334.23	0.19
0.1927	1347.99	1343.32	0.35	1347.99	1343.32	0.35	1334.45	1329.82	0.35
0.2941	1344.39	1336.61	0.58	1344.39	1336.61	0.58	1330.81	1323.12	0.58
0.4005	1339.29	1327.54	0.88	1339.29	1327.54	0.88	1325.63	1314.09	0.88
0.4996	1332.52	1316.5	1.22	1332.52	1316.5	1.22	1318.79	1303.09	1.20
0.6077	1321.25	1299.41	1.68	1321.25	1299.41	1.68	1307.43	1286.07	1.66
0.7064	1304.22	1276.09	2.20	1304.22	1276.09	2.20	1290.36	1262.91	2.17
0.8058	1273.04	1237.51	2.87	1273.04	1237.51	2.87	1259.29	1224.76	2.82
0.901	1209.25	1168.89	3.45	1209.25	1168.89	3.45	1196.21	1157.51	3.34
0.9497	1142.44	1107.24	3.18	1142.44	1107.24	3.18	1130.82	1097.68	3.02
0.9645	1112.06	1081.91	2.79	1112.06	1081.91	2.79	1101.35	1073.27	2.62
1	999.1	999.1	0.00	999.1	999.1	0.00	994.02	994.02	0.00

a Standard uncertainty: *X*_1_ is ±0.0001, *d* is ±0.00003 g·cm^–3^, and *U* is ±0.3 m·s^–1^.^33^

**Table 3:** Mean Percentage Deviation of the Computed Value of Density for Eight Binary Mixtures of Water and ILs

*T* (K)	water + [BMim][dca]	water + [BMpy][TfO]	water + [BMpyr][TfO]	water + [HMim][dca]
288.15	0.88	0.60	0.65	0.90
298.15	0.88	0.94	0.65	1.00
308.15	0.90	0.96	0.68	1.02

*T* (K)	water + [BMim][tfo]	water + [BMpyr][dca]	water + [EEpy][ESO_4_]	water + [Mpy][MSO_4_]
288.15	0.72	1.13	0.68	1.50
298.15	0.73	1.13	0.73	1.50
308.15	0.75	1.17	0.74	1.45

### 3.3 Estimation of Internal Pressure Using FST

**Figure 2:**
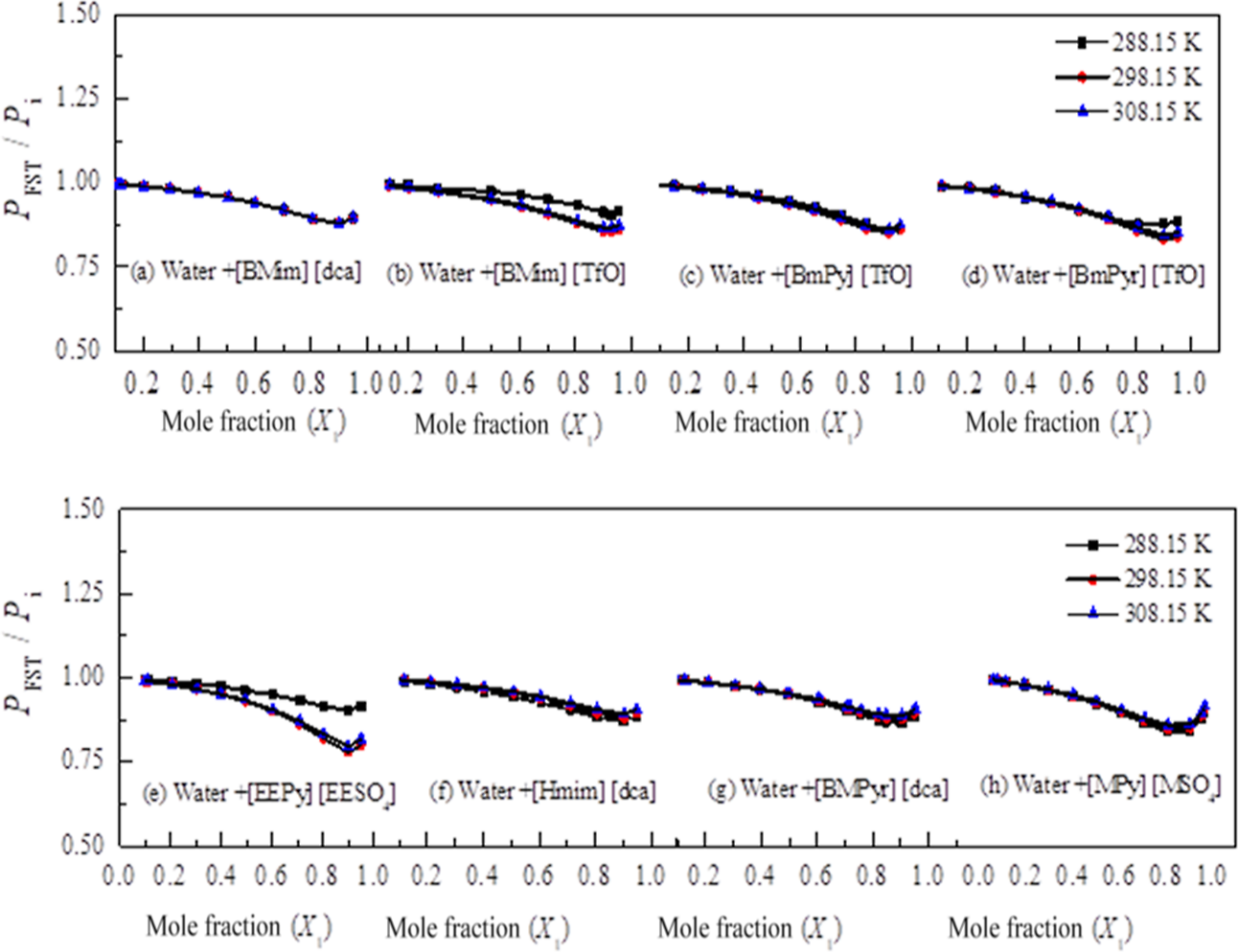
Ratio of internal pressure ratio (*P*_FST_/*P*_i_) obtained using the thermodynamic method and the FST method. Solid line is a guide for eye.

Internal pressure has been computed using the thermodynamic method (*P*_i_) and FST (*P*_FST_) for eight binary mixtures of water and ILs at various temperatures. The results obtained are reported in Table 4. Additionally, thermal expansivity and isothermal compressibility using the thermodynamic method (α, *K*_T_) and FST [α_FST_, (*K*_T_)_FST_] are computed and tabulated in Table S2 [Supporting file]. Due to the nonavailability of the experimental data of *P*i for these mixtures, the *P*i values obtained by using the thermodynamic method are considered standard to check the validity of FST in predicting the internal pressure. The ratio of *P*_FST_/*P*_i_ is plotted against the mole fraction (*X*_1_) for eight IL mixtures at different temperatures in Figure 2. As is obvious from Figure 2, the *P*_FST_/*P*_i_ ratio is closer to unity for all of the mixtures under study. It confirms the applicability of FST to predict the *P*i values for these mixtures. To generalize the validity of FST, this approach can be applied to these and similar ILs that are mixed with other suitable organic solvents.

**Table 4:** Internal Pressure Computed Using the Thermodynamic Approach (*P*_i_) and Flory’s Theory (*P*_FST_) at Different Temperatures for Eight IL Mixtures

*X*_1_	*P*_i_ (MPa)	P_FST_ (MPa)	*P*_i_ (MPa)	*P*_FST_ (MPa)	*P*_i_ (MPa)	*P*_FST_ (MPa)	*X*_1_	*P*_i_ (MPa)	P_FST_ (MPa)	*P*_i_ (MPa)	*P*_FST_ (MPa)	*P*_i_ (MPa)	*P*_FST_ (MPa)
	Water + [BMim][dca]							Water + [EEPy][ESO_4_]					
	*T* = 288.15 K		*T* = 298.15 K		*T* = 308.15 K			*T* = 288.15 K		*T* = 298.15 K		*T* = 308.15 K	
0	665.1	665.1	676.5	676.5	687.6	687.6	0	771.1	771	782.8	782.7	794.9	794.8
0.1088	663.2	659.7	675.1	671.5	686.5	682.9	0.0987	772.5	765.4	784.3	777.4	796.7	790
0.1227	663	658.9	675	670.8	686.3	682.2	0.1089	772.4	764.7	784.4	776.7	796.9	789.3
0.2017	661.9	654.3	673.9	666.5	685.3	678.2	0.2048	773.1	758.4	786.4	771	798.9	783.9
0.2943	660.6	648	672.6	660.6	684	672.7	0.3037	775.7	750	789.2	763.2	802	776.5
0.3952	658.8	639.6	671	652.7	682.5	665.4	0.3979	778.1	740.4	791.8	754.1	804.5	767.8
0.503	656.7	628.4	669.1	642.4	680.7	655.8	0.4922	781.6	728.3	795.4	742.8	808.3	757.1
0.5985	654.4	615.6	667	630.6	678.8	644.8	0.6018	786	709.4	799.9	724.9	812.5	740.3
0.7032	649.8	597.1	662.8	613.6	675	629.2	0.7084	791.3	683.5	805	700.5	817.5	717.2
0.805	638.8	571	652.8	589.9	665.7	607.7	0.8029	791.4	648.7	804.9	668.1	816.9	686.6
0.9006	607.8	534.5	625.5	557.9	641.8	579.8	0.8984	760.1	590.8	774.7	614.2	787.3	636.5
0.9503	567.4	507.7	590.6	535.5	612	561.5	0.9493	678.1	540.7	698.2	568.7	716.7	595.1
1	472.2	472.1	508.7	508.7	543.3	543.3	1	472.2	472.1	508.7	508.7	543.3	543.3
	Water + [BMim][TfO]							Water + [BmPy][TfO]					
	*T* = 288.15 K		*T* = 298.15 K		*T* = 308.15 K			*T* = 288.15 K		*T* = 298.15 K		*T* = 308.15 K	
0	585.2	585.1	593.9	593.9	602.1	602.1	0	589.4	589.4	597.9	598	606.2	606.2
0.1348	586.8	581.1	595.6	590.3	604	598.9	0.0794	587.1	590.4	596	599.2	604.5	607.2
0.2028	587.7	578.7	596.7	588.1	605.1	596.9	0.1517	584.8	591.5	593.9	600.3	602.6	608.4
0.2056	587.8	578.6	597.1	588.1	605.9	596.8	0.2502	581.1	593.3	590.5	602.2	599.5	610.5
0.3083	589.6	574.1	598.7	584.1	607.1	593.3	0.3515	576.4	595.3	586.4	604.6	595.8	613
0.4985	593.7	563.1	603.2	574.3	611.9	584.6	0.4528	570.6	598.1	581.2	607.5	591.2	616
0.6062	596.5	553.9	606.5	566.3	615.6	577.6	0.5628	562.5	601.9	574.1	611.5	584.9	620.2
	Water + [BMim][TfO]							Water + [BmPy][TfO]					
	*T* = 288.15 K		*T* = 298.15 K		*T* = 308.15 K			*T* = 288.15 K		*T* = 298.15 K		*T* = 308.15 K	
0.7015	599.2	543.2	609.9	557	619.4	569.7	0.6561	553.5	605.6	566.2	615.7	578	624.7
0.8064	599.7	527.2	612.6	543.7	623.8	558.9	0.7494	541.3	609.1	555.8	620.1	569.2	629.7
0.8998	590.7	505.6	608.1	526.9	623.4	546.6	0.8383	524.8	608.7	542.1	621.9	558.2	633.4
0.9309	580.8	497	601.1	521	619.3	543.3	0.9201	502.6	592.8	525.2	611.6	546.2	628.1
0.9548	567.6	489.3	591.1	516.2	612.2	541.3	0.9603	488.7	568.4	516.1	592.1	541.7	613.6
1	472.2	472.1	508.7	508.7	543.3	543.3	1	472.1	472.2	508.7	508.7	543.3	543.3
	Water + [BmPyr][TfO]							Water + [Hmim][dca]					
	*T* = 288.15 K		*T* = 298.15 K		*T* = 308.15 K			*T* = 288.15 K		*T* = 298.15 K		*T* = 308.15 K	
0	606.6	606.6	615.6	615.6	624.3	624.2	0	617.5	617.5	628	628	637.8	637.8
0.0559	607.4	604.8	616.6	614	625.6	622.8	0.0785	617.3	614.8	628	625.5	637.9	635.5
0.1084	608.2	603	617.4	612.4	626.1	621.4	0.1191	617.5	613.2	628	624	637.9	634.2
0.2076	610.2	599.2	619.6	609	628.4	618.2	0.2124	617.5	609.2	628.1	620.3	638	630.8
0.2997	612.5	595	622.1	605.2	631	614.7	0.3055	617.6	604.5	628.2	616	638.1	626.8
0.408	615.8	589	625.5	599.7	634.5	609.7	0.4019	617.6	598.5	628.3	610.5	638.2	621.8
0.5038	619.3	582.2	629.3	593.6	638.3	604.3	0.5096	617.7	590	628.5	602.8	638.6	614.7
0.6003	623.5	573.4	633.8	585.7	642.9	597.3	0.6069	617.6	580	628.6	593.7	638.5	606.5
0.7052	628.5	560.4	639.4	574.3	648.9	587.2	0.7123	616.1	565.2	627.3	580.3	637.3	594.4
0.8095	631.2	541.7	643.7	558.2	654.6	573.6	0.8067	610.3	546.1	622.5	563.5	633.3	579.6
0.9018	619.8	516.1	636.7	537.4	651.4	557.1	0.9031	587	516.9	603.5	538.8	618.3	559.3
0.9533	590.7	496	613.1	522.5	633.1	547.3	0.9499	556	497.4	578.7	523.9	599	548.6
	Water + [MPy][MSO_4_]							Water + [BMim][dca]					
	*T* = 288.15 K		*T* = 298.15 K		*T* = 308.15 K			*T* = 288.15 K		*T* = 298.15 K		*T* = 308.15 K	
0	943.6	943.5	963	963	982.6	982.6	0.3942	673.3	650.4	686.4	664.7	698.9	676.1
0.0574	941.4	936.9	961.4	956.5	980.7	976.3	0.4952	673.2	640.2	686.6	655.2	699.1	667.2
0.073	941.5	935	961.1	954.7	980.4	974.4	0.5023	673.3	639.4	686.6	654.4	699.1	666.5
0.1111	940.6	930.1	960.6	949.9	979.9	969.8	0.6047	673.4	625.8	686.9	641.7	699.5	654.8
0.1927	938.5	918.7	958.8	938.9	978.3	959.1	0.6137	673.3	624.4	686.8	640.4	699.4	653.6
0.2941	936.2	902	956.6	922.6	976	943.2	0.7061	671.4	607.3	685.1	624.6	697.8	639.1
0.4005	932.1	880.3	952.8	901.6	972.7	922.8	0.7177	670.9	604.7	684.7	622.2	697.4	636.9
0.4996	926.3	855.2	947.3	877.3	967.3	899.1	0.7571	668.0	595.1	682.1	613.3	694.9	628.9
0.6077	915	819.4	936.1	842.5	956.1	865.2	0.8208	658.3	575.6	673.1	595.6	686.6	612.9
0.7064	894	775.2	915.2	799.7	934.8	823.6	0.8468	651.1	565.9	666.6	586.8	678.6	605.1
0.8058	847.4	712.2	869.1	738.6	887.8	764.1	0.9034	623.7	540.0	642.0	563.8	658.7	585.0
0.901	740.1	621.9	764.6	651.4	786.7	679.6	0.904	623.2	539.6	641.4	563.5	658.3	584.8
0.9497	636	557.8	664.7	589.8	691.4	620.3	0.9492	579.8	512.4	603.2	540.2	624.6	565.6
0.9645	595	535	626.5	568.2	654.2	599.5	0.9507	577.8	511.4	601.5	539.4	623.2	564.9
1	472.2	472.1	508.7	508.7	543.3	543.3	0.9517	576.4	510.7	600.3	538.8	622.2	564.5
	Water + [BMim][dca]						1	472.2	472.1	508.7	508.7	543.3	543.3
	*T* = 288.15 K		*T* = 298.15 K		*T* = 308.15 K								
0	675.1	675.1	687.9	687.9	697.6	697.6							
0.096	674.5	670.6	687.4	683.6	697.7	693.6							
0.1277	674.3	668.9	687.2	682	698.1	692.2							
0.2116	673.9	664	686.9	677.5	698.3	687.9							
0.3077	673.9	657.5	686.6	671.3	698.2	682.2							

### 3.4 Thermophysical Parameters, Molecular Interactions, and FST

Some other important physical parameters, such as energy of vaporization (Δ*E*_V_), heat of vaporization (Δ*H*), cohesive energy density (ced), surface tension (σ), and polarity index (*n*), have been evaluated using FST in the given range of concentrations and temperatures. The obtained results of Δ*E*_V_, Δ*H*, and σ are reported in Table S3. In the current work, σ has been used in the calculation of the ultrasonic velocity using eqs 14–17. The importance of these parameters to understand the nature of molecular interactions prevalent in the various mixtures has been reported by many workers.^26,32,53−55^ The energy of vaporization (Δ*E*_V_) is defined as the energy utilized in the evaporation of one mol of the liquid by breaking all the associated forces, whereas the enthalpy of vaporization (Δ*H*) is a sum of the pressure-volume work done and the internal energy of the system. In the current study, these parameters are evaluated for given mixtures at given temperatures and concentrations, as reported in Table S1. The portrayal of Table S1 indicates that both Δ*E*_v_ and Δ*H*_v_ represent a similar decrease with an increase in the concentration of water in the mixtures. The decreasing trend in both of these parameters hints at a decrease in the cohesive forces with the addition of the first component.^56^ The ced represents both specific and nonspecific intermolecular interactions, which overall contribute to the total intermolecular interaction energies, whereas internal pressure counts only the specific interactions present in the liquid state. In the present study, ced is computed for eight IL mixtures, as reported in Table 5. The perusal of Table 5 indicates that ced decreases with a decrease in concentration of the ILs in the given mixtures at all temperatures. The decreasing values of ced suggest a decrease in the cohesion present within the liquid mixture and, hence, an increase in the molecular interactions.^57^ The Hildebrand solubility parameter (δ) is the square root of the ced and is important to access the intermolecular interactions in the liquid system. Several workers^58,59^ have calculated δ for organic liquid mixtures and polymer mixtures to analyze their solubility. Recently, Pandey and co-worker^32,50^ computed δ for pure ILs. In the present study, δ is calculated for given mixtures of water at various concentrations and temperatures, as reported in Table 5. The portrayal of Table 5 indicates that δ values gradually decrease in the water-rich region in these binary mixtures. This indicates that the solute–solute (A–A) interactions dominate over the solute–solvent (A–B) interactions in the water-rich region of the mixtures.

**Table 5:** Cohesive Energy Density (ced) and Solubility Parameters (δ) for Eight Binary Mixtures at Different Temperatures

*X*_1_	ced (kJ/m^3^)	δ (kJ/m^3^)^1/2^	ced (kJ/m^3^)	δ (kJ/m^3^)^1/2^	ced (kJ/m^3^)	δ (kJ/m^3^)^1/2^	*X*_1_	ced (kJ/m^3^)	δ (kJ/m^3^)^1/2^	ced (kJ/m^3^)	δ (kJ/m^3^)^1/2^	ced (kJ/m^3^)	δ (kJ/m^3^)^1/2^
	Water + [BMim][dca]							Water + [EEPy][ESO_4_]					
	*T* = 288.15 K		*T* = 298.15 K		*T* = 308.15 K			*T* = 288.15 K		*T* = 298.15 K		*T* = 308.15 K	
0	66.51	815.52	67.65	822.49	68.76	829.2	0	62.43	790.1	77.11	878.11	79.49	891.56
0.1088	66.54	815.74	67.69	822.74	68.8	829.47	0.0987	62.48	790.44	77.23	878.8	79.63	892.36
0.1227	66.55	815.76	67.69	822.76	68.81	829.51	0.1089	62.54	790.8	77.23	878.82	79.63	892.38
0.2017	66.57	815.93	67.72	822.95	68.84	829.73	0.2048	62.65	791.53	77.4	879.75	79.81	893.38
0.2943	66.59	816.06	67.75	823.13	68.88	829.96	0.3037	62.77	792.3	77.52	880.46	79.96	894.2
0.3952	66.59	816.05	67.77	823.2	68.91	830.11	0.3979	62.94	793.35	77.65	881.2	80.09	894.91
0.503	66.55	815.81	67.75	823.1	68.91	830.15	0.4922	63.11	794.42	77.74	881.73	80.21	895.6
0.5985	66.42	814.99	67.65	822.49	68.85	829.74	0.6018	63.3	795.59	77.71	881.55	80.22	895.66
0.7032	66.03	812.6	67.33	820.58	68.6	828.25	0.7084	63.47	796.71	77.34	879.4	79.93	894.06
0.805	64.91	805.64	66.37	814.66	67.78	823.29	0.8029	63.45	796.58	76.02	871.89	78.84	887.94
0.9006	61.75	785.83	63.63	797.71	65.44	808.92	0.8984	62.51	790.63	71.35	844.69	74.86	865.2
0.9503	57.5	758.27	59.92	774.08	62.22	788.8	0.9493	60.41	777.22	64.28	801.77	68.87	829.86
1	47.22	687.14	50.87	713.26	54.33	737.07	1	54.33	737.07	47.22	687.14	54.33	737.07
	Water + [BMim][TfO]							Water + [BmPy][TfO]					
	*T* = 288.15 K		*T* = 298.15 K		*T* = 308.15 K			*T* = 288.15 K		*T* = 298.15 K		*T* = 308.15 K	
0	58.52	764.95	59.39	770.68	60.21	775.95	0	58.94	767.71	59.8	773.28	60.62	778.6
0.1348	58.64	765.76	59.53	771.54	60.35	776.87	0.0794	59.02	768.24	59.87	773.76	60.7	779.09
0.2028	58.71	766.24	59.6	772.03	60.43	777.4	0.1517	59.1	768.77	59.94	774.18	60.77	779.53
0.2056	58.72	766.26	59.61	772.07	60.44	777.42	0.2502	59.22	769.51	60.03	774.77	60.86	780.15
0.3083	58.82	766.95	59.72	772.81	60.57	778.24	0.3515	59.35	770.4	60.14	775.5	60.98	780.93
0.4985	59.05	768.46	59.99	774.55	60.87	780.2	0.4528	59.5	771.37	60.27	776.34	61.13	781.84
0.6062	59.16	769.13	60.14	775.49	61.06	781.42	0.5628	59.68	772.53	60.43	777.36	61.31	782.99
	Water + [BMim][TfO]							Water + [BmPy][TfO]					
	*T* = 288.15 K		*T* = 298.15 K		*T* = 308.15 K			*T* = 288.15 K		*T* = 298.15 K		*T* = 308.15 K	
0.7015	59.17	769.25	60.23	776.1	61.23	782.47	0.6561	59.81	773.35	60.55	778.17	61.47	784.03
0.8064	58.91	767.55	60.15	775.59	61.32	783.05	0.7494	59.82	773.43	60.61	778.53	61.6	784.88
0.8998	57.42	757.74	59.1	768.75	60.67	778.93	0.8383	59.37	770.52	60.33	776.72	61.5	784.22
0.9309	56.16	749.4	58.14	762.53	60.01	774.66	0.9201	57.37	757.46	58.86	767.23	60.49	777.73
0.9548	54.48	738.11	56.82	753.78	59.02	768.24	0.9603	54.49	738.18	56.65	752.65	58.81	766.91
1	47.22	687.14	50.87	713.26	54.33	737.07	1	47.22	687.14	50.87	713.26	54.33	737.07
	Water + [BmPyr][TfO]							Water + [Hmim][dca]					
	*T* = 288.15 K		*T* = 298.15 K		*T* = 308.15 K			*T* = 288.15 K		*T* = 298.15 K		*T* = 308.15 K	
0	60.66	778.86	60.66	778.86	62.43	790.1	0	68.76	829.2	62.8	792.45	63.78	798.65
0.0559	60.7	779.11	60.7	779.11	62.48	790.44	0.0785	68.8	829.47	62.87	792.89	63.85	799.09
0.1084	60.75	779.43	60.75	779.43	62.54	790.8	0.1191	68.81	829.51	62.91	793.14	63.89	799.34
0.2076	60.86	780.12	60.86	780.12	62.65	791.53	0.2124	68.84	829.73	63.01	793.78	64	799.98
0.2997	60.97	780.83	60.97	780.83	62.77	792.3	0.3055	68.88	829.96	63.13	794.55	64.12	800.75
0.408	61.12	781.76	61.12	781.76	62.94	793.35	0.4019	68.91	830.11	63.28	795.5	64.27	801.7
0.5038	61.25	782.63	61.25	782.63	63.11	794.42	0.5096	68.91	830.15	63.49	796.79	64.48	803.01
0.6003	61.37	783.42	61.37	783.42	63.3	795.59	0.6069	68.85	829.74	63.71	798.19	64.72	804.46
0.7052	61.42	783.68	61.42	783.68	63.47	796.71	0.7123	68.6	828.25	63.96	799.74	64.99	806.16
0.8095	61.06	781.43	61.06	781.43	63.45	796.58	0.8067	67.78	823.29	64.06	800.4	65.17	807.29
0.9018	59.29	770	59.29	770	62.51	790.63	0.9031	65.44	808.92	63.28	795.49	64.68	804.26
0.9533	55.99	748.27	55.99	748.27	60.41	777.22	0.9499	62.22	788.8	61.2	782.33	63.06	794.1
1	47.22	687.14	47.22	687.14	54.33	737.07	1	54.33	737.07	50.87	713.26	54.33	737.07
	Water + [MPy][MSO_4_]							Water + [BMim][dca]					
	*T* = 288.15 K		*T* = 298.15 K		*T* = 308.15 K			*T* = 288.15 K		*T* = 298.15 K		*T* = 308.15 K	
0	94.36	971.38	94.36	971.38	98.26	991.26	0.3942	67.75	67.75	823.09	836.96	70.05	836.96
0.0574	94.33	971.24	94.33	971.24	98.23	991.11	0.4952	67.78	67.78	823.27	837.34	70.11	837.34
0.073	94.32	971.17	94.32	971.17	98.22	991.04	0.5023	67.78	67.78	823.27	837.36	70.12	837.36
0.1111	94.29	971.02	94.29	971.02	98.18	990.88	0.6047	67.72	67.72	822.94	837.4	70.12	837.4
0.1927	94.21	970.63	94.21	970.63	98.11	990.5	0.6137	67.71	67.71	822.86	837.36	70.12	837.36
0.2941	94.04	969.76	94.04	969.76	97.94	989.66	0.7061	67.42	67.42	821.1	836.3	69.94	836.3
0.4005	93.72	968.08	93.72	968.08	97.63	988.08	0.7177	67.35	67.35	820.69	836.03	69.89	836.03
0.4996	93.2	965.39	93.2	965.39	97.14	985.57	0.7571	67.04	67.04	818.8	834.71	69.67	834.71
0.6077	92.11	959.72	92.11	959.72	96.09	980.27	0.8208	66.1	66.1	813.01	830.5	68.97	830.5
0.7064	90.16	949.55	90.16	949.55	94.25	970.85	0.8468	65.44	65.44	808.97	827.51	68.48	827.51
0.8058	86.02	927.48	86.02	927.48	90.35	950.51	0.9034	62.96	62.96	793.48	816	66.58	816
0.901	76.7	875.76	76.7	875.76	81.62	903.46	0.904	62.92	62.92	793.23	815.81	66.56	815.81
0.9497	66.83	817.51	66.83	817.51	72.47	851.28	0.9492	58.72	58.72	766.32	795.77	63.33	795.77
0.9645	62.46	790.34	62.46	790.34	68.43	827.21	0.9507	58.52	58.52	765.01	794.79	63.17	794.79
1	47.22	687.14	47.22	687.14	54.33	737.07	0.9517	58.39	58.39	764.11	794.12	63.06	794.12
	Water + [BMim][dca]						1	47.22	684.11	54.33	737.07	54.33	737.07
	*T* = 288.15 K		*T* = 298.15 K		*T* = 308.15 K								
0	67.51	821.67	67.51	821.67	69.76	835.25							
0.096	67.57	822.01	67.57	822.01	69.83	835.62							
0.1277	67.59	822.13	67.59	822.13	69.85	835.75							
0.2116	67.64	822.44	67.64	822.44	69.91	836.12							
0.3077	67.7	822.78	67.7	822.78	69.98	836.55							

## 4 Conclusions

In the current study, density, internal pressure, ultrasonic velocity, and some important thermophysical properties of eight binary mixtures of water and ILs have been evaluated at three different temperatures using FST. The very low mean percentage deviation of computed density values (Table 3) and the *P*_FST_/*P*_i_ ratio being closer to unity (Figure 2) for all the mixtures under study confirm the applicability of FST for the evaluation of density and internal pressure of the liquid mixtures. The reasonable agreement of the Auerbach relation (*U*_A_), Singh–Pandey–Sanguri relation (*U*_SP_), and modified Auerbach relation (*U*_MA_) with the literature values (U*) validates the *U*–*d*–*σ* correlation for the given systems. The variations of the solubility parameter (δ) in the given concentration at different temperatures indicate the dominance of A–A interactions over A–B in the water-rich region of the water + IL mixtures.

## Supporting Information Available

Computed values of ultrasonic velocity using Auerbach (*U*_A_), Alternberg (*U*_AR_), Singh–Pandey–Sanguri (*U*_SP_), and modified Auerbach (*U*_MA_) relations; volume expansivity (α) and isothermal compressibility (*K*_T_); and internal pressure (*P*_i_ and *P*_FST_), energy of vaporization (Δ*E*_V_), heat of vaporization (Δ*H*), surface tension (σ) for eight binary mixtures of water, and ILs at different temperatures (PDF)
