# Supplementary material for: Estimation of Ultrasonic Velocity, Density, Internal Pressure, and Thermophysical Parameters of Ionic Liquid Mixtures: Application of Flory’s Statistical Theory
Source: ACS Omega. 2024 Apr 22;9(17):19363–77. doi: 10.1021/acsomega.4c00520 (PMC11064050; doi:10.1021/acsomega.4c00520)
Supplement: Supplementary file 1 — ao4c00520_si_001.pdf [file ao4c00520_si_001.pdf]

Estimation of Ultrasonic Velocity, Density, Internal Pressure, and Thermo-Physical Parameters of Ionic Liquid Mixtures: Application of Flory's Statistical Theory

Archana Sirohi[1], Arun Upmanyu[1*], Pankaj Kumar[1], Monika Dhiman[1], Kailash Chandra Juglan[2], Devinder Pal Singh[3], Kuldeep K Saxena[4], Alok Bhadauria[5*], Md Irfanul Haque Siddiqui[6]

[1]Chitkara University Institute of Engineering and Technology, Chitkara University, Punjab, 140401, India; [2]Department of Physics, Lovely Faculty of Technology and Sciences, Lovely Professional University, Punjab, India; [3]Acoustics Research Center, Mississauga, L5A 1Y7, Ontario, Canada; [4]Division of Research and Development, Lovely Professional University, Phagwara, Punjab, 144001, India; [5]Department of Mechanical and Industrial Engineering, Manipal Institute of Technology Bengaluru, Manipal Academy of Higher Education, Manipal, India; [6]Mechanical Engineering Department, College of Engineering, King Saud University, Riyadh 11451, Saudi Arabia

*Corresponding Author Email: alok.bhadauria@manipal.edu, arun.upmanyu@chikara.edu.in

Table S1: Computed values of ultrasonic velocity using Auerbach (U_A), Atterberg (U_{AR}), Singh – Pandey –Sanguri (U_{SP}), and modified Auerbach (U_{MA})relations at different temperatures.

X_1	U_A	U_{AR}	U_{SP}	U_{MA}
**Water + [BMim][dca]**				
**T=288.15K**				
0	1721.413	1896.940	1668.003	1840.188
0.1088	1724.828	1934.826	1671.313	1843.839
0.1227	1725.371	1940.137	1671.838	1844.419
0.2017	1728.539	1972.106	1674.908	1847.805
0.2943	1732.996	2014.662	1679.227	1852.570
0.3952	1738.851	2068.724	1684.900	1858.830
0.503	1746.805	2138.956	1692.607	1867.332
0.5985	1756.032	2216.493	1701.548	1877.196
0.7032	1768.968	2325.504	1714.082	1891.024
0.805	1785.555	2469.111	1730.155	1908.756
0.9006	1802.509	2656.727	1746.583	1926.879
0.9503	1807.440	2779.796	1751.361	1932.151
1	1792.268	2894.130	1736.660	1915.932
**T=298.15K**				
X_1	U_A	U_{AR}	U_{SP}	U_{MA}
0	1739.698	1919.515	1672.990	1859.735
0.1088	1743.381	1958.210	1676.531	1863.672
0.1227	1743.923	1963.572	1677.052	1864.251
0.2017	1747.127	1995.887	1680.133	1867.676
0.2943	1751.602	2038.868	1684.436	1872.460
0.3952	1757.594	2093.633	1690.199	1878.865
0.503	1765.617	2164.582	1697.914	1887.442
0.5985	1774.827	2242.706	1706.771	1897.288
0.7032	1787.684	2352.421	1719.135	1911.031
0.805	1803.892	2496.444	1734.722	1928.358
0.9006	1819.868	2684.225	1750.085	1945.436
0.9503	1823.712	2807.654	1753.782	1949.546
1	1807.185	2926.307	1737.889	1931.878
**T=308.15K**				
X_1	U_A	U_{AR}	U_{SP}	U_{MA}
0	1757.588	1941.420	1677.847	1878.859
0.1088	1761.423	1980.704	1681.509	1882.959
0.1227	1761.940	1986.075	1682.002	1883.511
0.2017	1765.232	2018.823	1685.145	1887.031
0.2943	1769.728	2062.193	1689.437	1891.837
0.3952	1775.800	2117.550	1695.234	1898.328
0.503	1783.914	2189.206	1702.979	1907.001
0.5985	1793.188	2268.020	1711.832	1916.915
0.7032	1806.044	2378.490	1724.105	1930.658
0.805	1821.946	2522.946	1739.286	1947.658
0.9006	1837.225	2711.263	1753.871	1963.990
0.9503	1840.244	2835.311	1756.754	1967.219
1	1822.814	2958.361	1740.114	1948.586
**Water + [BMim][TfO]**				
**T=288.15K**				
X_1	U_A	U_{AR}	U_{SP}	U_{MA}
0	1501.193	1672.087	1454.616	1604.774
0.1348	1508.173	1718.715	1461.379	1612.235
0.2028	1512.363	1745.725	1465.439	1616.714
0.2056	1512.587	1746.967	1465.656	1616.953
0.3083	1520.357	1793.959	1473.185	1625.259
0.4985	1540.570	1908.079	1492.771	1646.867
0.6062	1557.829	1997.760	1509.495	1665.317
0.7015	1579.345	2103.160	1530.343	1688.318
0.8064	1614.575	2266.784	1564.480	1725.978

0.8998	1669.326	2494.678	1617.532	1784.507
0.9309	1697.099	2601.196	1644.444	1814.197
0.9548	1725.542	2700.449	1672.004	1844.602
1	1792.268	2894.130	1736.660	1915.932
		**T=298.15K**		
X_1	U_A	U_{AR}	U_{SP}	U_{MA}
0	1517.012	1691.694	1458.843	1621.684
0.1348	1524.073	1738.852	1465.632	1629.232
0.2028	1528.425	1766.332	1469.817	1633.884
0.2056	1528.749	1767.777	1470.129	1634.231
0.3083	1536.573	1815.195	1477.653	1642.594
0.4985	1557.144	1930.773	1497.435	1664.584
0.6062	1574.697	2021.601	1514.315	1683.349
0.7015	1596.465	2128.213	1535.249	1706.619
0.8064	1632.150	2293.943	1569.565	1744.766
0.8998	1686.845	2523.849	1622.163	1803.235
0.9309	1714.254	2630.850	1648.520	1832.535
0.9548	1741.994	2729.926	1675.197	1862.189
1	1807.185	2926.307	1737.889	1931.878
		**T=308.15K**		
X_1	U_A	U_{AR}	U_{SP}	U_{MA}
0	1532.538	1710.735	1463.007	1638.280
0.1348	1539.823	1758.654	1469.963	1646.069
0.2028	1544.201	1786.373	1474.142	1650.748
0.2056	1544.715	1788.140	1474.632	1651.298
0.3083	1552.485	1835.817	1482.050	1659.604
0.4985	1573.417	1952.782	1502.032	1681.980
0.6062	1591.216	2044.645	1519.024	1701.008
0.7015	1613.235	2152.349	1540.044	1724.546
0.8064	1649.332	2319.965	1574.503	1763.134
0.8998	1704.155	2551.978	1626.839	1821.739
0.9309	1731.366	2659.697	1652.815	1850.827
0.9548	1758.580	2758.842	1678.794	1879.919
1	1822.814	2958.361	1740.114	1948.586
		**Water + [HMim][dca]**		
		**T=288.15K**		
X_1	U_A	U_{AR}	U_{SP}	U_{MA}
0	1755.419	1873.943	1700.954	1876.540
0.0785	1757.832	1901.148	1703.292	1879.120
0.1191	1759.315	1916.470	1704.729	1880.706
0.2124	1763.005	1954.834	1708.304	1884.650
0.3055	1767.396	1998.802	1712.559	1889.343
0.4019	1772.872	2052.032	1717.865	1895.197
0.5096	1780.556	2124.251	1725.311	1903.412
0.6069	1789.389	2205.870	1733.870	1912.855
0.7123	1801.724	2320.802	1745.822	1926.040
0.8067	1815.257	2460.196	1758.936	1940.508
0.9031	1827.366	2658.280	1770.669	1953.452
0.9499	1825.455	2779.325	1768.817	1951.409
1	1792.268	2894.130	1736.660	1915.932
		**T=298.15K**		
X_1	U_A	U_{AR}	U_{SP}	U_{MA}
0	1773.926	1896.044	1705.905	1896.324
0.0785	1776.483	1923.722	1708.364	1899.058
0.1191	1777.934	1939.134	1709.759	1900.609
0.2124	1781.693	1977.961	1713.374	1904.627
0.3055	1786.149	2022.437	1717.659	1909.391
0.4019	1791.694	2076.242	1722.991	1915.318
0.5096	1799.501	2149.254	1730.499	1923.664
0.6069	1808.431	2231.670	1739.087	1933.210

0.7123	1820.720	2347.389	1750.905	1946.347
0.8067	1834.065	2487.578	1763.737	1960.612
0.9031	1845.630	2687.062	1774.859	1972.976
0.9499	1843.044	2809.597	1772.373	1970.212
1	1807.185	2926.307	1737.889	1931.878
**T=308.15K**				
X_1	U_A	U_{AR}	U_{SP}	U_{MA}
0	1792.059	1917.497	1710.755	1915.709
0.0785	1794.655	1945.481	1713.233	1918.484
0.1191	1796.125	1961.060	1714.636	1920.055
0.2124	1799.971	2000.350	1718.308	1924.166
0.3055	1804.483	2045.290	1722.615	1928.990
0.4019	1810.119	2099.674	1727.995	1935.015
0.5096	1818.076	2173.498	1735.591	1943.520
0.6069	1827.031	2256.548	1744.140	1953.094
0.7123	1839.319	2373.040	1755.871	1966.230
0.8067	1852.578	2514.113	1768.528	1980.403
0.9031	1863.816	2715.193	1779.256	1992.416
0.9499	1860.658	2839.075	1776.241	1989.040
1	1822.814	2958.361	1740.114	1948.586
**Water+ [BMpy][TfO]**				
**T=288.15K**				
X_1	U_A	U_{AR}	U_{SP}	U_{MA}
0	1570.510	1732.467	1499.257	1678.873
0.0559	1573.452	1751.840	1502.066	1682.018
0.1084	1576.112	1770.780	1504.605	1684.862
0.2076	1582.402	1811.419	1510.610	1691.586
0.2997	1589.466	1855.149	1517.354	1699.137
0.408	1599.764	1916.337	1527.184	1710.145
0.5038	1611.439	1982.660	1538.329	1722.626
0.6003	1626.806	2066.208	1552.999	1739.054
0.7052	1650.017	2186.450	1575.157	1763.866
0.8095	1684.754	2357.758	1608.318	1800.999
0.9018	1734.386	2589.548	1655.698	1854.056
0.9533	1777.538	2777.263	1696.893	1900.186
1	1822.814	2958.361	1740.114	1948.586
**T=298.15K**				
X_1	U_A	U_{AR}	U_{SP}	U_{MA}
0	1532.994	1700.696	1474.212	1638.769
0.0794	1536.838	1727.634	1477.908	1642.878
0.1517	1540.863	1754.726	1481.779	1647.180
0.2502	1547.505	1796.779	1488.166	1654.280
0.3515	1555.824	1847.567	1496.166	1663.173
0.4528	1566.377	1908.878	1506.314	1674.455
0.5628	1581.603	1992.705	1520.957	1690.732
0.6561	1599.251	2084.865	1537.928	1709.597
0.7494	1623.987	2207.861	1561.716	1736.040
0.8383	1659.317	2373.349	1595.691	1773.808
0.9201	1712.346	2601.745	1646.686	1830.495
0.9603	1754.516	2761.883	1687.239	1875.575
1	1807.185	2926.307	1737.889	1931.878
**T=308.15K**				
X_1	U_A	U_{AR}	U_{SP}	U_{MA}
0	1548.532	1719.765	1478.277	1655.379
0.0794	1552.371	1746.894	1481.941	1659.483
0.1517	1556.503	1774.362	1485.886	1663.900
0.2502	1563.289	1816.970	1492.364	1671.153
0.3515	1571.784	1868.406	1500.473	1680.234
0.4528	1582.526	1930.461	1510.728	1691.718
0.5628	1597.965	2015.166	1525.467	1708.223

0.6561	1615.858	2108.311	1542.548	1727.350
0.7494	1640.825	2232.460	1566.382	1754.040
0.8383	1676.384	2399.516	1600.328	1792.052
0.9201	1729.380	2629.884	1650.920	1848.705
0.9603	1770.687	2790.150	1690.352	1892.862
1	1822.814	2958.361	1740.114	1948.586
**Water+ [BMpyr][TfO]**				
**T=288.15K**				
X_1	U_A	U_{AR}	U_{SP}	U_{MA}
0	1538.920	1690.560	1491.173	1645.104
0.0559	1541.621	1709.131	1493.789	1647.991
0.1084	1544.302	1727.769	1496.387	1650.857
0.2076	1550.258	1767.227	1502.158	1657.224
0.2997	1556.986	1809.758	1508.678	1664.416
0.408	1566.885	1869.408	1518.269	1674.998
0.5038	1578.183	1934.175	1529.217	1687.075
0.6003	1593.126	2015.893	1543.697	1703.050
0.7052	1615.885	2133.840	1565.749	1727.378
0.8095	1650.167	2302.128	1598.968	1764.027
0.9018	1700.116	2531.109	1647.367	1817.421
0.9533	1745.431	2719.599	1691.276	1865.863
1	1792.268	2894.130	1736.660	1915.932
**T=298.15K**				
X_1	U_A	U_{AR}	U_{SP}	U_{MA}
0	1554.922	1710.314	1495.299	1662.209
0.0559	1557.688	1729.240	1497.958	1665.166
0.1084	1560.390	1748.107	1500.557	1668.054
0.2076	1566.531	1788.158	1506.462	1674.619
0.2997	1573.449	1831.314	1513.115	1682.014
0.408	1583.552	1891.735	1522.831	1692.815
0.5038	1595.047	1957.287	1533.885	1705.103
0.6003	1610.236	2039.982	1548.491	1721.340
0.7052	1633.232	2159.147	1570.605	1745.922
0.8095	1667.754	2329.080	1603.804	1782.827
0.9018	1717.552	2559.835	1651.693	1836.061
0.9533	1761.621	2748.112	1694.072	1883.170
1	1807.185	2926.307	1737.889	1931.878
**T=308.15K**				
X_1	U_A	U_{AR}	U_{SP}	U_{MA}
0	1570.510	1729.440	1499.257	1678.873
0.0559	1573.451	1748.789	1502.065	1682.017
0.1084	1576.110	1767.706	1504.603	1684.859
0.2076	1582.399	1808.299	1510.607	1691.582
0.2997	1589.463	1851.987	1517.351	1699.134
0.408	1599.764	1913.128	1527.184	1710.146
0.5038	1611.448	1979.418	1538.338	1722.635
0.6003	1626.833	2062.957	1553.025	1739.082
0.7052	1650.083	2183.257	1575.220	1763.937
0.8095	1684.904	2354.815	1608.462	1801.160
0.9018	1734.674	2587.312	1655.974	1854.364
0.9533	1777.885	2775.936	1697.224	1900.557
1	1822.814	2958.361	1740.114	1948.586
**Water+ [BMpyr][dca]**				
**T=288.15K**				
X_1	U_A	U_{AR}	U_{SP}	U_{MA}
0	1774.609	1921.656	1719.549	1897.055
0.096	1777.453	1955.649	1722.304	1900.095
0.1277	1778.483	1967.797	1723.302	1901.195
0.2116	1781.592	2002.716	1726.315	1904.519

0.3077	1785.997	2048.730	1730.583	1909.228
0.3942	1790.449	2096.374	1734.897	1913.987
0.4952	1797.106	2163.071	1741.347	1921.104
0.5023	1797.653	2168.331	1741.877	1921.688
0.6047	1806.514	2254.120	1750.464	1931.161
0.6137	1807.360	2262.624	1751.284	1932.065
0.7061	1817.382	2363.264	1760.994	1942.779
0.7177	1818.759	2377.776	1762.329	1944.251
0.7571	1823.556	2430.841	1766.976	1949.378
0.8208	1831.303	2531.160	1774.484	1957.661
0.8468	1834.016	2578.018	1777.113	1960.561
0.9034	1836.909	2693.753	1779.916	1963.653
0.904	1836.887	2695.044	1779.895	1963.630
0.9492	1831.157	2799.397	1774.342	1957.505
0.9507	1830.731	2802.943	1773.930	1957.049
0.9517	1830.425	2805.273	1773.633	1956.722
1	1792.268	2894.130	1736.660	1915.932
		**T=298.15K**		
X_1	U_A	U_{AR}	U_{SP}	U_{MA}
0	1792.939	1944.299	1724.188	1916.649
0.096	1795.878	1978.761	1727.015	1919.791
0.1277	1796.938	1991.072	1728.034	1920.924
0.2116	1800.114	2026.419	1731.088	1924.319
0.3077	1804.445	2072.715	1735.253	1928.949
0.3942	1809.081	2121.074	1739.711	1933.905
0.4952	1815.880	2188.570	1746.250	1941.173
0.5023	1816.429	2193.871	1746.778	1941.760
0.6047	1825.290	2280.299	1755.300	1951.233
0.6137	1826.170	2288.934	1756.146	1952.173
0.7061	1836.103	2390.125	1765.697	1962.791
0.7177	1837.465	2404.728	1767.008	1964.248
0.7571	1842.171	2458.012	1771.533	1969.278
0.8208	1849.586	2558.502	1778.664	1977.205
0.8468	1852.090	2605.430	1781.072	1979.882
0.9034	1854.312	2721.349	1783.208	1982.256
0.904	1854.189	2722.414	1783.090	1982.125
0.9492	1847.571	2827.450	1776.726	1975.050
0.9507	1847.122	2831.062	1776.294	1974.571
0.9517	1846.759	2833.365	1775.945	1974.183
1	1807.185	2926.307	1737.889	1931.878
		**T=308.15K**		
X_1	U_A	U_{AR}	U_{SP}	U_{MA}
0	1810.809	1966.164	1728.654	1935.752
0.096	1814.020	2001.387	1731.720	1939.185
0.1277	1815.325	2014.199	1732.965	1940.580
0.2116	1818.769	2050.327	1736.253	1944.261
0.3077	1823.262	2097.300	1740.542	1949.065
0.3942	1828.309	2146.783	1745.360	1954.459
0.4952	1835.187	2214.974	1751.926	1961.812
0.5023	1835.714	2220.274	1752.429	1962.375
0.6047	1844.656	2307.515	1760.966	1971.935
0.6137	1845.535	2316.201	1761.805	1972.874
0.7061	1855.360	2417.920	1771.184	1983.378
0.7177	1856.737	2432.642	1772.498	1984.849
0.7571	1861.336	2486.129	1776.889	1989.766
0.8208	1868.448	2586.844	1783.678	1997.368
0.8468	1869.871	2631.930	1785.036	1998.890
0.9034	1872.370	2749.952	1787.422	2001.561
0.904	1872.330	2751.250	1787.384	2001.519
0.9492	1864.687	2856.344	1780.088	1993.348

0.9507	1864.227	2860.050	1779.649	1992.856
0.9517	1863.860	2862.413	1779.298	1992.463
1	1822.814	2958.361	1740.114	1948.586
		**Water + [EEpy][ESO_4]**		
		**T=288.15K**		
X_1	U_A	U_{AR}	U_{SP}	U_{MA}
0	1570.582	1771.911	1521.852	1678.950
0.0987	1575.091	1806.535	1526.221	1683.770
0.1089	1575.599	1810.306	1526.713	1684.313
0.2048	1580.389	1848.595	1531.355	1689.433
0.3037	1587.329	1895.549	1538.079	1696.852
0.3979	1595.238	1948.021	1545.743	1705.308
0.4922	1605.596	2011.725	1555.780	1716.380
0.6018	1621.875	2105.399	1571.553	1733.782
0.7084	1645.132	2229.184	1594.088	1758.643
0.8029	1676.662	2383.950	1624.640	1792.349
0.8984	1727.801	2614.338	1674.193	1847.017
0.9493	1763.608	2767.942	1708.889	1885.295
1	1792.268	2894.130	1736.660	1915.932
		**T=298.15K**		
X_1	U_A	U_{AR}	U_{SP}	U_{MA}
0	1586.849	1792.977	1526.001	1696.339
0.0987	1591.442	1828.059	1530.418	1701.249
0.1089	1592.010	1831.946	1530.964	1701.856
0.2048	1597.099	1871.255	1535.858	1707.296
0.3037	1604.129	1918.791	1542.619	1714.812
0.3979	1612.214	1971.916	1550.394	1723.455
0.4922	1622.593	2036.198	1560.375	1734.550
0.6018	1638.905	2130.528	1576.061	1751.987
0.7084	1662.015	2254.689	1598.285	1776.692
0.8029	1692.958	2409.015	1628.041	1809.769
0.8984	1742.459	2636.984	1675.644	1862.686
0.9493	1776.753	2789.405	1708.624	1899.347
1	1807.185	2926.307	1737.889	1931.878
		**T=308.15K**		
X_1	U_A	U_{AR}	U_{SP}	U_{MA}
0	1602.749	1813.409	1530.034	1713.336
0.0987	1607.329	1848.901	1534.405	1718.232
0.1089	1607.946	1852.907	1534.995	1718.892
0.2048	1613.179	1892.696	1539.990	1724.486
0.3037	1620.313	1940.852	1546.801	1732.112
0.3979	1628.465	1994.431	1554.582	1740.826
0.4922	1638.954	2059.353	1564.596	1752.040
0.6018	1655.220	2154.152	1580.124	1769.428
0.7084	1678.300	2278.822	1602.157	1794.101
0.8029	1708.766	2432.793	1631.241	1826.669
0.8984	1756.878	2658.714	1677.170	1878.100
0.9493	1790.314	2811.125	1709.089	1913.843
1	1822.814	2958.361	1740.114	1948.586
		**Water + [Mpy][MSO_4]**		
		**T=288.15K**		
X_1	U_A	U_{AR}	U_{SP}	U_{MA}
0	1472.589	1758.728	1426.900	1574.196
0.0574	1475.547	1777.719	1429.766	1577.358
0.073	1476.608	1783.469	1430.794	1578.492
0.1111	1478.979	1797.447	1433.091	1581.026
0.1927	1484.649	1830.022	1438.585	1587.088
0.2941	1493.438	1877.015	1447.102	1596.483
0.4005	1504.967	1935.560	1458.273	1608.808

0.4996	1518.826	2002.088	1471.702	1623.623
0.6077	1539.360	2093.713	1491.598	1645.573
0.7064	1565.636	2203.110	1517.060	1673.663
0.8058	1604.833	2352.676	1555.041	1715.565
0.901	1664.558	2556.220	1612.912	1779.410
0.9497	1712.029	2697.731	1658.910	1830.156
0.9645	1730.954	2748.953	1677.248	1850.388
1	1792.268	2894.130	1736.660	1915.932
		**T=298.15K**		
X_1	U_A	U_{AR}	U_{SP}	U_{MA}
0	1487.731	1779.986	1430.684	1590.382
0.0574	1490.830	1799.388	1433.664	1593.695
0.073	1491.809	1805.027	1434.605	1594.742
0.1111	1494.269	1819.284	1436.972	1597.372
0.1927	1500.022	1852.279	1442.504	1603.522
0.2941	1508.816	1899.648	1450.960	1612.922
0.4005	1520.399	1958.750	1462.100	1625.305
0.4996	1534.265	2025.780	1475.434	1640.127
0.6077	1554.667	2117.742	1495.053	1661.937
0.7064	1580.632	2227.148	1520.022	1689.693
0.8058	1619.235	2376.264	1557.145	1730.960
0.901	1678.074	2579.176	1613.728	1793.859
0.9497	1725.372	2721.818	1659.213	1844.421
0.9645	1744.872	2775.208	1677.964	1865.265
1	1807.185	2926.307	1737.889	1931.878
		**T=308.15K**		
X_1	U_A	U_{AR}	U_{SP}	U_{MA}
0	1502.564	1800.628	1434.394	1606.239
0.0574	1505.607	1820.086	1437.299	1609.492
0.073	1506.583	1825.774	1438.231	1610.535
0.1111	1509.056	1840.154	1440.591	1613.179
0.1927	1514.868	1873.519	1446.139	1619.391
0.2941	1523.663	1921.248	1454.536	1628.794
0.4005	1535.341	1980.957	1465.684	1641.277
0.4996	1549.216	2048.447	1478.929	1656.110
0.6077	1569.547	2140.824	1498.338	1677.844
0.7064	1595.183	2250.140	1522.811	1705.248
0.8058	1632.993	2398.254	1558.906	1745.667
0.901	1691.490	2601.485	1614.748	1808.200
0.9497	1739.097	2746.043	1660.196	1859.093
0.9645	1758.458	2799.727	1678.679	1879.790
1	1822.814	2958.361	1740.114	1948.586

Table S2. Volume expansivity (α) and Isothermal compressibility (K_T) computed using thermodynamic approach (P_i) and Flory's theory (P_{FST})] at different temperatures

X_1	$\alpha \times 10^{-4}$	$K_T \times 10^{-10}$	*Flory's parameters*	
			$\alpha_{FST} \times 10^{-4}$	$K_{FST} \times 10^{-10}$
Water + [BMim][dca]				
		T =288.15 K		
0	9.389	4.068	9.389	4.068
0.1088	9.398	4.084	9.401	4.106
0.1227	9.399	4.085	9.402	4.112
0.2017	9.404	4.094	9.41	4.144
0.2943	9.41	4.104	9.421	4.189
0.3952	9.419	4.12	9.435	4.251
0.503	9.429	4.138	9.451	4.334
0.5985	9.44	4.157	9.469	4.432
0.7032	9.462	4.196	9.497	4.583

X_1	$\alpha \times 10^{-4}$	$K_T \times 10^{-10}$	Flory's parameters	
			$\alpha_{FST} \times 10^{-4}$	$K_{FST} \times 10^{-10}$
0.805	9.516	4.293	9.561	4.825
0.9006	9.675	4.587	9.72	5.24
0.9503	9.9	5.028	9.935	5.639
1	10.525	6.423	10.524	6.423
		T =298.15 K		
0	9.443	4.162	9.442	4.162
0.1088	9.449	4.173	9.453	4.197
0.1227	9.45	4.174	9.455	4.203
0.2017	9.454	4.183	9.463	4.233
0.2943	9.46	4.193	9.474	4.276
0.3952	9.468	4.207	9.488	4.334
0.503	9.477	4.223	9.505	4.411
0.5985	9.487	4.241	9.524	4.503
0.7032	9.507	4.276	9.552	4.642
0.805	9.555	4.364	9.614	4.859
0.9006	9.692	4.62	9.753	5.212
0.9503	9.88	4.987	9.928	5.528
1	10.384	6.085	10.383	6.085
		T=308.15 K		
0	9.495	4.256	9.495	4.256
0.1088	9.5	4.265	9.505	4.289
0.1227	9.501	4.266	9.507	4.294
0.2017	9.506	4.274	9.516	4.324
0.2943	9.512	4.285	9.527	4.364
0.3952	9.519	4.298	9.542	4.419
0.503	9.527	4.313	9.56	4.492
0.5985	9.536	4.329	9.579	4.577
0.7032	9.554	4.362	9.609	4.706
0.805	9.598	4.443	9.669	4.903
0.9006	9.716	4.665	9.79	5.204
0.9503	9.871	4.97	9.932	5.45
1	10.271	5.826	10.271	5.826
Water + [BMim][TfO]				
		T=288.15 K		
0	9.798	4.825	9.798	4.825
0.1348	9.789	4.807	9.793	4.856
0.2028	9.784	4.797	9.789	4.875
0.2056	9.783	4.796	9.788	4.875
0.3083	9.774	4.777	9.784	4.91
0.4985	9.751	4.732	9.77	5
0.6062	9.736	4.703	9.762	5.078
0.7015	9.721	4.675	9.754	5.174
0.8064	9.719	4.67	9.752	5.33
0.8998	9.767	4.765	9.804	5.587
0.9309	9.823	4.874	9.852	5.712
0.9548	9.898	5.025	9.926	5.845
1	10.525	6.423	10.524	6.423
		T=298.15 K		
0	9.861	4.95	9.861	4.95
0.1348	9.852	4.932	9.856	4.978
0.2028	9.846	4.92	9.853	4.995
0.2056	9.844	4.915	9.851	4.994
0.3083	9.835	4.898	9.848	5.027
0.4985	9.81	4.849	9.835	5.106
0.6062	9.792	4.814	9.827	5.174
0.7015	9.774	4.778	9.818	5.255
0.8064	9.76	4.75	9.807	5.378

X_1	α × 10^{-4}	K_T× 10^{-10}	*Flory's parameters*	
			α_{FST} × 10^{-4}	K_{FST}× 10^{-10}
0.8998	9.784	4.797	9.836	5.566
0.9309	9.822	4.871	9.866	5.646
0.9548	9.877	4.982	9.918	5.728
1	10.384	6.085	10.383	6.085
		T=308.15 K		
0	9.925	5.08	9.925	5.08
0.1348	9.914	5.058	9.919	5.104
0.2028	9.909	5.046	9.917	5.119
0.2056	9.904	5.037	9.913	5.118
0.3083	9.897	5.023	9.913	5.148
0.4985	9.871	4.971	9.901	5.219
0.6062	9.852	4.932	9.894	5.278
0.7015	9.832	4.891	9.885	5.347
0.8064	9.808	4.845	9.869	5.441
0.8998	9.811	4.849	9.877	5.569
0.9309	9.832	4.892	9.89	5.61
0.9548	9.87	4.968	9.922	5.648
1	10.271	5.826	10.271	5.826
Water + [BmPy][TfO]				
		T=288.15 K		
0	9.775	4.779	9.774	4.779
0.0794	9.769	4.767	9.77	4.795
0.1517	9.763	4.757	9.766	4.812
0.2502	9.753	4.737	9.76	4.84
0.3515	9.742	4.715	9.752	4.875
0.4528	9.727	4.686	9.742	4.919
0.5628	9.707	4.647	9.726	4.982
0.6561	9.687	4.609	9.709	5.055
0.7494	9.668	4.573	9.693	5.16
0.8383	9.67	4.578	9.697	5.324
0.9201	9.756	4.742	9.783	5.609
0.9603	9.894	5.015	9.92	5.849
1	10.525	6.423	10.524	6.423
		T=298.15 K		
0	9.839	4.906	9.839	4.906
0.0794	9.832	4.893	9.834	4.919
0.1517	9.826	4.881	9.83	4.935
0.2502	9.816	4.86	9.824	4.96
0.3515	9.803	4.835	9.816	4.991
0.4528	9.787	4.804	9.807	5.031
0.5628	9.766	4.761	9.791	5.085
0.6561	9.744	4.718	9.775	5.148
0.7494	9.721	4.674	9.757	5.235
0.8383	9.711	4.656	9.753	5.364
0.9201	9.766	4.761	9.808	5.568
0.9603	9.871	4.97	9.91	5.725
1	10.384	6.085	10.383	6.085
		T=308.15 K		
0	9.902	5.034	9.902	5.034
0.0794	9.897	5.022	9.898	5.046
0.1517	9.89	5.009	9.895	5.06
0.2502	9.879	4.986	9.889	5.083
0.3515	9.866	4.959	9.882	5.111
0.4528	9.849	4.927	9.873	5.146
0.5628	9.827	4.882	9.859	5.194
0.6561	9.804	4.836	9.844	5.248
0.7494	9.778	4.785	9.826	5.319

X_1	α × 10^{-4}	K_T× 10^{-10}	*Flory's parameters*	
			α_{FST} × 10^{-4}	K_{FST}× 10^{-10}
0.8383	9.759	4.748	9.814	5.417
0.9201	9.786	4.802	9.843	5.553
0.9603	9.863	4.953	9.911	5.638
1	10.271	5.826	10.271	5.826
Water + [BmPyr][TfO]				
		T=288.15 K		
0	9.681	4.599	9.681	4.599
0.0559	9.677	4.591	9.679	4.612
0.1084	9.673	4.583	9.677	4.624
0.2076	9.662	4.563	9.67	4.65
0.2997	9.65	4.54	9.661	4.678
0.408	9.633	4.508	9.648	4.72
0.5038	9.615	4.473	9.634	4.768
0.6003	9.593	4.433	9.617	4.833
0.7052	9.567	4.386	9.596	4.934
0.8095	9.554	4.361	9.587	5.1
0.9018	9.612	4.469	9.648	5.387
0.9533	9.767	4.765	9.804	5.696
1	10.525	6.423	10.524	6.423
		T=298.15 K		
0	9.744	4.719	9.744	4.719
0.0559	9.739	4.709	9.74	4.73
0.1084	9.734	4.701	9.738	4.741
0.2076	9.723	4.679	9.731	4.764
0.2997	9.71	4.654	9.723	4.79
0.408	9.692	4.62	9.711	4.828
0.5038	9.673	4.583	9.698	4.871
0.6003	9.65	4.54	9.682	4.928
0.7052	9.622	4.487	9.662	5.016
0.8095	9.6	4.447	9.647	5.153
0.9018	9.635	4.512	9.687	5.375
0.9533	9.757	4.745	9.807	5.596
1	10.384	6.085	10.383	6.085
		T=308.15 K		
0	9.806	4.841	9.806	4.841
0.0559	9.799	4.827	9.802	4.85
0.1084	9.796	4.821	9.801	4.86
0.2076	9.784	4.798	9.794	4.882
0.2997	9.771	4.772	9.787	4.906
0.408	9.753	4.737	9.776	4.941
0.5038	9.734	4.699	9.765	4.98
0.6003	9.71	4.654	9.75	5.03
0.7052	9.68	4.597	9.731	5.106
0.8095	9.652	4.544	9.713	5.218
0.9018	9.668	4.573	9.735	5.385
0.9533	9.76	4.75	9.821	5.53
1	10.271	5.826	10.271	5.826
Water + [EEPy][ESO_4]				
		T=288.15 K		
0	8.937	3.34	8.937	3.34
0.0987	8.932	3.332	8.931	3.362
0.1089	8.932	3.332	8.932	3.366
0.2048	8.929	3.328	8.924	3.391
0.3037	8.92	3.313	8.915	3.425
0.3979	8.91	3.3	8.903	3.465
0.4922	8.897	3.28	8.889	3.517
0.6018	8.88	3.256	8.871	3.603

X_1	α × 10^{-4}	K_T× 10^{-10}	*Flory's parameters*	
			α_{FST} × 10^{-4}	K_{FST}× 10^{-10}
0.7084	8.861	3.227	8.851	3.732
0.8029	8.86	3.226	8.857	3.934
0.8984	8.98	3.404	9.003	4.391
0.9493	9.328	3.964	9.384	5.001
1	10.525	6.423	10.524	6.423
		T=298.15 K		
0	8.994	3.426	8.994	3.426
0.0987	8.988	3.417	8.988	3.447
0.1089	8.988	3.416	8.988	3.45
0.2048	8.98	3.405	8.976	3.471
0.3037	8.97	3.389	8.966	3.503
0.3979	8.96	3.374	8.956	3.541
0.4922	8.946	3.353	8.941	3.589
0.6018	8.93	3.328	8.926	3.671
0.7084	8.911	3.3	8.91	3.792
0.8029	8.911	3.301	8.918	3.98
0.8984	9.026	3.474	9.062	4.398
0.9493	9.343	3.99	9.408	4.932
1	10.384	6.085	10.383	6.085
		T=308.15 K		
0	9.047	3.507	9.047	3.507
0.0987	9.041	3.497	9.039	3.526
0.1089	9.04	3.496	9.039	3.529
0.2048	9.032	3.484	9.028	3.549
0.3037	9.02	3.466	9.017	3.578
0.3979	9.011	3.451	9.008	3.615
0.4922	8.997	3.43	8.995	3.661
0.6018	8.981	3.406	8.982	3.739
0.7084	8.963	3.379	8.969	3.854
0.8029	8.965	3.382	8.982	4.031
0.8984	9.076	3.552	9.123	4.417
0.9493	9.365	4.027	9.436	4.887
1	10.271	5.826	10.271	5.826
Water + [MPy][MSO_4]				
		T=288.15 K		
0	8.356	2.552	8.355	2.552
0.0574	8.362	2.56	8.363	2.572
0.073	8.362	2.559	8.364	2.578
0.1111	8.364	2.562	8.368	2.592
0.1927	8.371	2.57	8.376	2.627
0.2941	8.377	2.578	8.388	2.68
0.4005	8.39	2.594	8.406	2.751
0.4996	8.407	2.615	8.429	2.84
0.6077	8.442	2.659	8.473	2.98
0.7064	8.507	2.742	8.549	3.178
0.8058	8.66	2.945	8.721	3.528
0.901	9.06	3.528	9.14	4.235
0.9497	9.53	4.317	9.602	4.961
0.9645	9.744	4.719	9.806	5.281
1	10.525	6.423	10.524	6.423
		T=298.15 K		
0	8.394	2.599	8.394	2.599
0.0574	8.399	2.605	8.4	2.618
0.073	8.399	2.606	8.402	2.624
0.1111	8.401	2.608	8.405	2.638
0.1927	8.406	2.614	8.413	2.672
0.2941	8.413	2.622	8.425	2.723

X_1	$\alpha \times 10^{-4}$	$K_T \times 10^{-10}$	*Flory's parameters*	
			$\alpha_{FST} \times 10^{-4}$	$K_{FST} \times 10^{-10}$
0.4005	8.424	2.636	8.442	2.792
0.4996	8.44	2.656	8.465	2.877
0.6077	8.474	2.699	8.509	3.011
0.7064	8.538	2.781	8.585	3.201
0.8058	8.686	2.98	8.753	3.533
0.901	9.065	3.535	9.152	4.189
0.9497	9.498	4.26	9.576	4.841
0.9645	9.687	4.61	9.755	5.119
1	10.384	6.085	10.383	6.085
		T=308.15 K		
0	8.43	2.644	8.43	2.644
0.0574	8.435	2.651	8.437	2.663
0.073	8.436	2.651	8.438	2.668
0.1111	8.438	2.654	8.442	2.682
0.1927	8.442	2.659	8.45	2.715
0.2941	8.449	2.667	8.462	2.764
0.4005	8.459	2.68	8.478	2.831
0.4996	8.474	2.7	8.501	2.914
0.6077	8.507	2.742	8.545	3.043
0.7064	8.571	2.826	8.622	3.226
0.8058	8.72	3.026	8.789	3.544
0.901	9.078	3.556	9.169	4.158
0.9497	9.478	4.224	9.56	4.749
0.9645	9.654	4.547	9.724	4.998
1	10.271	5.826	10.271	5.826
Water + [HMim][dca]				
		T=288.15 K		
0	9.624	4.491	9.624	4.491
0.0785	9.625	4.492	9.626	4.512
0.1191	9.624	4.491	9.627	4.524
0.2124	9.624	4.491	9.629	4.555
0.3055	9.624	4.49	9.632	4.592
0.4019	9.623	4.49	9.635	4.639
0.5096	9.623	4.489	9.639	4.707
0.6069	9.623	4.49	9.644	4.791
0.7123	9.631	4.504	9.658	4.924
0.8067	9.662	4.561	9.694	5.115
0.9031	9.788	4.804	9.822	5.476
0.9499	9.966	5.165	9.992	5.788
1	10.525	6.423	10.524	6.423
		T=298.15 K		
0	9.68	4.596	9.679	4.596
0.0785	9.679	4.595	9.682	4.615
0.1191	9.679	4.595	9.683	4.626
0.2124	9.679	4.595	9.686	4.655
0.3055	9.679	4.594	9.689	4.69
0.4019	9.678	4.593	9.693	4.734
0.5096	9.677	4.59	9.698	4.797
0.6069	9.677	4.59	9.704	4.873
0.7123	9.683	4.603	9.72	4.994
0.8067	9.708	4.65	9.755	5.161
0.9031	9.809	4.846	9.86	5.456
0.9499	9.947	5.125	9.988	5.684
1	10.384	6.085	10.383	6.085
		T=308.15 K		
0	9.736	4.704	9.736	4.704
0.0785	9.736	4.703	9.738	4.722

X_1	$\alpha \times 10^{-4}$	$K_T \times 10^{-10}$	*Flory's parameters*	
			$\alpha_{FST} \times 10^{-4}$	$K_{FST} \times 10^{-10}$
0.1191	9.736	4.703	9.74	4.732
0.2124	9.735	4.702	9.743	4.76
0.3055	9.735	4.701	9.747	4.792
0.4019	9.734	4.7	9.752	4.833
0.5096	9.732	4.696	9.758	4.892
0.6069	9.733	4.697	9.767	4.962
0.7123	9.739	4.709	9.785	5.072
0.8067	9.759	4.748	9.818	5.22
0.9031	9.837	4.902	9.903	5.457
0.9499	9.942	5.115	9.996	5.615
1	10.271	5.826	10.271	5.826
Water + [BmPyr][dca]				
		T=288.15 K		
0	9.342	3.987	9.342	3.987
0.096	9.345	3.992	9.347	4.017
0.1277	9.346	3.994	9.349	4.027
0.2116	9.348	3.997	9.353	4.059
0.3077	9.348	3.997	9.357	4.101
0.3942	9.35	4.002	9.361	4.147
0.4952	9.351	4.002	9.365	4.215
0.5023	9.351	4.002	9.365	4.221
0.6047	9.35	4.001	9.368	4.314
0.6137	9.35	4.002	9.369	4.324
0.7061	9.359	4.016	9.382	4.452
0.7177	9.362	4.021	9.385	4.472
0.7571	9.375	4.044	9.401	4.552
0.8208	9.421	4.124	9.451	4.731
0.8468	9.456	4.185	9.488	4.831
0.9034	9.592	4.432	9.626	5.137
0.904	9.594	4.436	9.629	5.141
0.9492	9.828	4.885	9.859	5.544
0.9507	9.84	4.907	9.87	5.561
0.9517	9.847	4.923	9.878	5.573
1	10.525	6.423	10.524	6.423
		T=298.15 K		
0	9.39	4.07	9.39	4.07
0.096	9.392	4.074	9.395	4.098
0.1277	9.393	4.075	9.397	4.108
0.2116	9.395	4.078	9.401	4.138
0.3077	9.396	4.08	9.407	4.178
0.3942	9.397	4.082	9.411	4.221
0.4952	9.396	4.08	9.415	4.285
0.5023	9.396	4.08	9.416	4.29
0.6047	9.395	4.078	9.42	4.377
0.6137	9.395	4.078	9.421	4.386
0.7061	9.403	4.092	9.436	4.504
0.7177	9.405	4.095	9.439	4.523
0.7571	9.417	4.116	9.454	4.596
0.8208	9.458	4.189	9.503	4.757
0.8468	9.489	4.245	9.536	4.845
0.9034	9.609	4.462	9.659	5.108
0.904	9.612	4.468	9.662	5.113
0.9492	9.81	4.849	9.855	5.439
0.9507	9.82	4.867	9.864	5.453
0.9517	9.826	4.881	9.87	5.462
1	10.384	6.085	10.383	6.085
		T=308.15 K		

X_1	$\alpha \times 10^{-4}$	$K_T \times 10^{-10}$	*Flory's parameters*	
			$\alpha_{FST} \times 10^{-4}$	$K_{FST} \times 10^{-10}$
0	9.449	4.174	9.449	4.174
0.096	9.449	4.173	9.453	4.199
0.1277	9.448	4.171	9.453	4.208
0.2116	9.447	4.169	9.456	4.236
0.3077	9.447	4.169	9.461	4.274
0.3942	9.444	4.164	9.463	4.313
0.4952	9.443	4.162	9.469	4.373
0.5023	9.443	4.162	9.469	4.378
0.6047	9.441	4.159	9.475	4.459
0.6137	9.441	4.16	9.477	4.468
0.7061	9.449	4.173	9.493	4.577
0.7177	9.451	4.176	9.496	4.594
0.7571	9.462	4.196	9.512	4.661
0.8208	9.5	4.263	9.559	4.806
0.8468	9.537	4.33	9.597	4.888
0.9034	9.632	4.506	9.698	5.108
0.904	9.634	4.509	9.7	5.111
0.9492	9.804	4.837	9.862	5.373
0.9507	9.811	4.851	9.869	5.383
0.9517	9.817	4.862	9.874	5.39
1	10.271	5.826	10.271	5.826

Table S3. Thermophysical parameters for eight binary mixtures of water and ionic liquids at different temperatures

X_1	σ	ΔE_v (KJ)	ΔH_v (KJ)	σ	ΔE_v (KJ)	ΔH_v (KJ)	σ	ΔE_v (KJ)	ΔH_v (KJ)
Water +[BMim][dca]									
	T=288.15K			T=298.15K			T=308.15K		
0	45.29	127.99	130.39	45.22	130.98	133.45	45.13	133.92	136.48
0.1088	45.36	115.46	117.85	45.29	118.16	120.64	45.21	120.83	123.39
0.1227	45.38	113.85	116.25	45.31	116.52	119	45.23	119.15	121.72
0.2017	45.44	104.74	107.13	45.37	107.2	109.68	45.29	109.63	112.2
0.2943	45.52	94.03	96.42	45.46	96.25	98.73	45.38	98.45	101.02
0.3952	45.61	82.32	84.71	45.56	84.28	86.76	45.49	86.23	88.8
0.503	45.71	69.75	72.15	45.66	71.45	73.93	45.61	73.13	75.69
0.5985	45.76	58.54	60.94	45.74	60	62.48	45.7	61.45	64.01
0.7032	45.7	46.12	48.52	45.72	47.33	49.81	45.73	48.53	51.1
0.805	45.23	33.79	36.19	45.35	34.78	37.26	45.45	35.75	38.32
0.9006	43.4	21.83	24.23	43.8	22.64	25.12	44.15	23.43	25.99
0.9503	40.65	15.34	17.74	41.43	16.07	18.55	42.11	16.78	19.34
1	33.6	8.51	10.91	35.27	9.19	11.67	36.75	9.85	12.41
Water +[BMim][TfO]									
	T=288.15K			T=298.15K			T=308.15K		
0	40.51	129.02	131.42	40.37	131.76	134.24	40.2	134.39	136.95
0.1348	40.66	113.29	115.68	40.51	115.71	118.19	40.35	118.04	120.6
0.2028	40.74	105.34	107.74	40.6	107.6	110.08	40.44	109.78	112.34
0.2056	40.74	105.01	107.41	40.61	107.27	109.75	40.45	109.44	112
0.3083	40.89	92.98	95.37	40.75	94.99	97.47	40.59	96.93	99.5
0.4985	41.21	70.59	72.99	41.1	72.17	74.65	40.96	73.7	76.26
0.6062	41.42	57.81	60.21	41.33	59.15	61.63	41.22	60.45	63.01
0.7015	41.59	46.4	48.8	41.55	47.54	50.02	41.47	48.64	51.2
0.8064	41.6	33.66	36.05	41.67	34.59	37.07	41.7	35.49	38.05
0.8998	40.83	21.94	24.34	41.18	22.72	25.2	41.47	23.47	26.04
0.9309	40.02	17.92	20.32	40.57	18.66	21.14	41.03	19.37	21.93
0.9548	38.94	14.75	17.15	39.71	15.47	17.95	40.38	16.15	18.72
1	33.6	8.51	10.91	35.27	9.19	11.67	36.75	9.85	12.41

X_1	σ	ΔE_v (KJ)	ΔH_v (KJ)	σ	ΔE_v (KJ)	ΔH_v (KJ)	σ	ΔE_v (KJ)	ΔH_v (KJ)
Water + [BMpy][TfO]									
	T=288.15K				T=298.15K			T=308.15K	
0	40.77	133.41	135.8	40.6	156.46	158.94	40.44	159.57	162.13
0.0794	40.85	123.82	126.21	40.69	145.09	147.56	40.52	147.98	150.54
0.1517	40.94	115.08	117.47	40.78	134.71	137.19	40.61	137.4	139.97
0.2502	41.07	103.14	105.54	40.92	120.55	123.03	40.76	122.98	125.54
0.3515	41.22	90.84	93.23	41.09	105.97	108.45	40.93	108.12	110.68
0.4528	41.41	78.5	80.89	41.3	91.35	93.83	41.15	93.23	95.79
0.5628	41.64	65.03	67.43	41.57	75.41	77.89	41.43	77	79.56
0.6561	41.84	53.53	55.92	41.84	61.81	64.29	41.72	63.15	65.72
0.7494	41.99	41.89	44.28	42.11	48.09	50.57	42.03	49.2	51.76
0.8383	41.86	30.57	32.96	42.21	34.79	37.27	42.25	35.7	38.26
0.9201	40.7	19.78	22.18	41.52	22.19	24.67	41.84	22.95	25.51
0.9603	38.85	14.25	16.65	40.06	15.78	18.26	40.71	16.47	19.03
1	33.6	8.51	10.91	35.27	9.19	11.67	36.75	9.85	12.41
Water +[BMpyr][dca]									
	T=288.15K				T=298.15K			T=308.15K	
0	45.89	138.65	141.05	45.89	138.65	141.05	45.71	144.87	147.44
0.096	45.97	126.62	129.02	45.97	126.62	129.02	45.8	132.32	134.88
0.1277	46	122.65	125.04	46	122.65	125.04	45.83	128.17	130.73
0.2116	46.08	112.11	114.51	46.08	112.11	114.51	45.93	117.18	119.75
0.3077	46.2	100.02	102.41	46.2	100.02	102.41	46.05	104.57	107.13
0.3942	46.3	89.1	91.5	46.3	89.1	91.5	46.17	93.19	95.75
0.4952	46.43	76.3	78.7	46.43	76.3	78.7	46.33	79.86	82.42
0.5023	46.44	75.4	77.8	46.44	75.4	77.8	46.34	78.92	81.48
0.6047	46.56	62.32	64.72	46.56	62.32	64.72	46.49	65.31	67.87
0.6137	46.57	61.17	63.56	46.57	61.17	63.56	46.5	64.1	66.67
0.7061	46.57	49.22	51.61	46.57	49.22	51.61	46.57	51.69	54.25
0.7177	46.55	47.7	50.1	46.55	47.7	50.1	46.56	50.12	52.68
0.7571	46.45	42.53	44.93	46.45	42.53	44.93	46.52	44.75	47.32
0.8208	46.01	34.05	36.44	46.01	34.05	36.44	46.23	35.98	38.54
0.8468	45.66	30.53	32.92	45.66	30.53	32.92	45.95	32.35	34.91
0.9034	44.18	22.71	25.1	44.18	22.71	25.1	44.89	24.3	26.87
0.904	44.15	22.62	25.02	44.15	22.62	25.02	44.87	24.22	26.78
0.9492	41.45	16.15	18.55	41.45	16.15	18.55	42.82	17.6	20.17
0.9507	41.32	15.93	18.33	41.32	15.93	18.33	42.72	17.38	19.94
0.9517	41.23	15.79	18.18	41.23	15.79	18.18	42.65	17.23	19.79
1	33.6	8.51	10.91	33.6	8.51	10.91	36.75	9.85	12.41
Water + [BMpyr][TfO]									
	T=288.15K				T=298.15K			T=308.15K	
0	41.8	140.22	142.62	41.8	140.22	142.62	41.48	145.99	148.55
0.0559	41.86	133.09	135.49	41.86	133.09	135.49	41.55	138.58	141.15
0.1084	41.93	126.4	128.8	41.93	126.4	128.8	41.61	131.63	134.19
0.2076	42.06	113.75	116.14	42.06	113.75	116.14	41.75	118.48	121.04
0.2997	42.2	101.98	104.37	42.2	101.98	104.37	41.9	106.25	108.81
0.408	42.4	88.1	90.49	42.4	88.1	90.49	42.11	91.83	94.39
0.5038	42.6	75.77	78.17	42.6	75.77	78.17	42.33	79.03	81.6
0.6003	42.82	63.28	65.68	42.82	63.28	65.68	42.58	66.08	68.65
0.7052	43.04	49.57	51.97	43.04	49.57	51.97	42.89	51.89	54.46
0.8095	43.06	35.7	38.09	43.06	35.7	38.09	43.11	37.58	40.14
0.9018	42.15	23.01	25.4	42.15	23.01	25.4	42.71	24.56	27.12
0.9533	40.06	15.6	18	40.06	15.6	18	41.37	17.02	19.58
1	33.6	8.51	10.91	33.6	8.51	10.91	36.75	9.85	12.41
Water+[EEpy][ESO4]									
	T=288.15K				T=298.15K			T=308.15K	
0	41.48	145.99	148.55	51.52	163.75	166.14	51.21	170.65	173.21
0.0559	41.55	138.58	141.15	51.65	149.13	151.53	51.34	155.45	158.01

X_1	σ	ΔE_v (KJ)	ΔH_v (KJ)	σ	ΔE_v (KJ)	ΔH_v (KJ)	σ	ΔE_v (KJ)	ΔH_v (KJ)
0.1084	41.61	131.63	134.19	51.66	147.61	150.01	51.36	153.86	156.42
0.2076	41.75	118.48	121.04	51.79	133.41	135.81	51.5	139.08	141.64
0.2997	41.9	106.25	108.81	51.96	118.68	121.08	51.68	123.75	126.31
0.408	42.11	91.83	94.39	52.13	104.61	107	51.85	109.09	111.65
0.5038	42.33	79.03	81.6	52.32	90.43	92.83	52.05	94.35	96.91
0.6003	42.58	66.08	68.65	52.52	73.8	76.2	52.26	77.06	79.62
0.7052	42.89	51.89	54.46	52.59	57.39	59.79	52.39	60.03	62.59
0.8095	43.11	37.58	40.14	52.2	42.47	44.87	52.12	44.59	47.15
0.9018	42.71	24.56	27.12	49.91	26.72	29.12	50.26	28.38	30.94
0.9533	41.37	17.02	19.58	45.61	17.83	20.23	46.7	19.31	21.87
1	36.75	9.85	12.41	33.6	8.51	10.91	36.75	9.85	12.41
Water+[HMim][dca]									
	T=288.15K				T=298.15K			T=308.15K	
0	45.13	133.92	136.48	42.38	231.57	234.05	42.27	236.63	239.19
0.1088	45.21	120.83	123.39	42.45	214.56	217.04	42.34	219.25	221.81
0.1227	45.23	119.15	121.72	42.49	205.76	208.24	42.39	210.25	212.82
0.2017	45.29	109.63	112.2	42.6	185.52	187.99	42.5	189.57	192.13
0.2943	45.38	98.45	101.02	42.73	165.3	167.78	42.63	168.92	171.48
0.3952	45.49	86.23	88.8	42.9	144.33	146.81	42.79	147.5	150.06
0.503	45.61	73.13	75.69	43.12	120.84	123.32	43.02	123.51	126.07
0.5985	45.7	61.45	64.01	43.38	99.54	102.02	43.28	101.76	104.32
0.7032	45.73	48.53	51.1	43.71	76.29	78.77	43.62	78.03	80.59
0.805	45.45	35.75	38.32	43.97	55.2	57.68	43.93	56.53	59.09
0.9006	44.15	23.43	25.99	43.66	33.09	35.56	43.81	34.04	36.6
0.9503	42.11	16.78	19.34	42.33	21.9	24.38	42.77	22.7	25.26
1	36.75	9.85	12.41	35.27	9.19	11.67	33.6	8.51	10.91
Water+ [Mpy][MSO_4]									
	T=288.15K				T=298.15K			T=308.15K	
0	61.41	143.14	145.54	61.41	143.14	145.54	61.6	150.56	153.12
0.0574	61.44	135.86	138.25	61.44	135.86	138.25	61.63	142.9	145.46
0.073	61.46	133.87	136.27	61.46	133.87	136.27	61.65	140.82	143.38
0.1111	61.49	129.03	131.42	61.49	129.03	131.42	61.68	135.72	138.28
0.1927	61.56	118.63	121.03	61.56	118.63	121.03	61.75	124.8	127.36
0.2941	61.63	105.66	108.05	61.63	105.66	108.05	61.82	111.16	113.72
0.4005	61.66	91.95	94.35	61.66	91.95	94.35	61.86	96.77	99.33
0.4996	61.6	79.07	81.47	61.6	79.07	81.47	61.81	83.26	85.82
0.6077	61.3	64.83	67.23	61.3	64.83	67.23	61.54	68.34	70.9
0.7064	60.53	51.57	53.96	60.53	51.57	53.96	60.83	54.47	57.03
0.8058	58.48	37.8	40.19	58.48	37.8	40.19	58.95	40.11	42.67
0.901	53.05	23.98	26.38	53.05	23.98	26.38	54.06	25.77	28.34
0.9497	46.75	16.56	18.95	46.75	16.56	18.95	48.43	18.11	20.67
0.9645	43.87	14.24	16.63	43.87	14.24	16.63	45.87	15.72	18.28
1	33.6	8.51	10.91	33.6	8.51	10.91	36.75	9.85	12.41
